# Novel β-Benzyloxy-Substituted Copolymers of Seven-Membered Cyclic Carbonate: Ring-Opening Polymerization with L-Lactide, ε-Caprolactone and Trimethylenecarbonate

**DOI:** 10.3390/polym16233364

**Published:** 2024-11-29

**Authors:** Valeriia A. Serova, Badma N. Mankaev, Milana U. Agaeva, Elena V. Chernikova, Anna K. Berkovich, Roman S. Alekseyev, Aleksei V. Khvostov, Sergey V. Timofeev, Sergey S. Karlov

**Affiliations:** 1 Chemistry Department, Lomonosov Moscow State University, Leninskye Gory 1, 119991 Moscow, Russia; valeriia.serova@chemistry.msu.ru (V.A.S.); mankaev@org.chem.msu.ru (B.N.M.); milaneagayeva@gmail.com (M.U.A.); chernikova_elena@mail.ru (E.V.C.); berkovichak@my.msu.ru (A.K.B.); alekseevrs@my.msu.ru (R.S.A.); alekse98@mail.ru (A.V.K.); timofeev@ineos.ac.ru (S.V.T.); 2 A.N. Nesmeyanov Institute of Organoelement Compounds, Russian Academy of Sciences, 28 Vavilov Str., 119991 Moscow, Russia; 3 Institute of Chemistry, Saint Petersburg State University, Universitetskaya Nab. 7/9, 199034 Saint Petersburg, Russia

**Keywords:** ring-opening polymerization, carbonate, lactide, ε-caprolactone, copolymers

## Abstract

To prepare novel biodegradable copolymers with functional substituents that are distributed statistically or randomly over the macromolecule chain and have improved characteristics compared to homopolymers, we conducted a series of synthetic experiments with a novel cyclic monomer, 5-(benzyloxy)-1,3-dioxepan-2-one (**4**). This compound was synthesized, and its homopolymer, as well as its copolymers with L-lactide, ε-caprolactone and trimethylene carbonate, were prepared in a polymerization solution with stannous octoate as the initiator. The formation of the copolymers was confirmed using NMR spectroscopy and DSC data. The distribution of the monomeric units of the substituted 7CC in the copolymers with L-lactide and ε-caprolactone is random, as it is close to a statistical distribution. The copolymer with TMC is a gradient copolymer due to the different rates of monomer polymerization. The copolymer with a composition of 10(ε-CL):1(carbonate **4**) can be considered a promising polymer after the deprotection of the hydroxy group for the inoculation of the functional substituents due to its convenience of preparation and properties similar to those of poly(ε-caprolactone).

## 1. Introduction

In the past three decades, biodegradable polymers such as polylactide (PLA), poly(ε-caprolactone) (PCL), and poly(trimethylene carbonate) (TMC) have become attractive alternatives to classic polyolefins in several fields of technology [1]. One of the most significant areas is the use of biodegradable polymers in medical applications due to their ability to decompose relatively quickly in living organisms, such as their use in suture material, tissue engineering, materials for controlled drug delivery, slow-release medication administration, bone surgery, orthopedics, the formation of artificial organs, nerve regeneration and wound healing [2,3]. The most convenient and frequently used method to synthesize these biodegradable polymers is ring-opening polymerization (ROP) of the cyclic monomer under the action of an initiator [4,5,6,7,8].

The chemical (degradation rate), physical and mechanical properties of the above-mentioned biodegradable polymers, as well as a number of their drawbacks, are well known; for example, the drawbacks of PLA include brittleness, poor elasticity, low thermal stability and poor gas/water permeability [9]. There are two different approaches used to improve some of these properties: (i) modification of the polymer via plasticization or blending, and (ii) ring-opening copolymerization (ROCOP) of the monomer with another comonomer [9,10]. The preparation of statistical copolymers with different ratios of comonomers may lead to biodegradable materials with controlled improved properties. In addition, it should be noted that the use of functionally substituted cyclic esters as one of the monomers in ROCOP leads to copolymers containing a certain number of functional groups that are suitable for further functionalization and are also capable of more rapid degradation.

Poly(alkylene carbonates) are biodegradable elastomers that exhibit high extensibility and the ability to recover their shape after deformation, as well as the ability to decompose into non-acidic and non-toxic remnants. The combination of polyesters and polycarbonates allows the creation of materials with good mechanical characteristics and degradation profiles. The use of cyclic carbonate monomers with various substituents can vary the thermal, mechanical and hydrophilic properties of these copolymers and gives them additional functionality [11,12].

**Figure 1:**
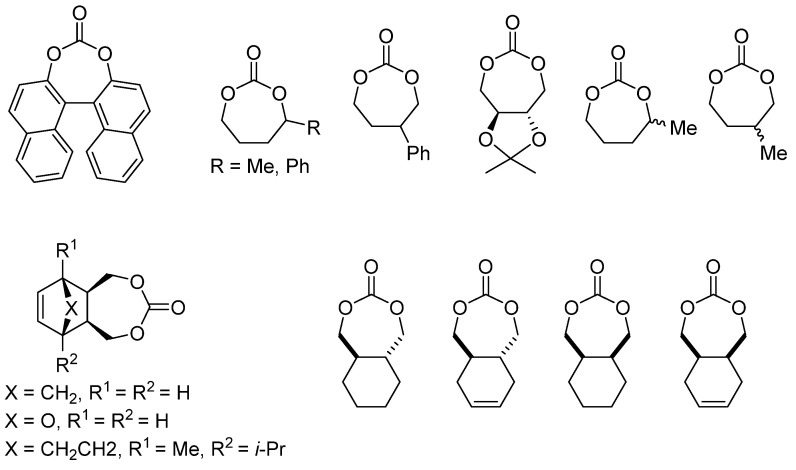
Previously studied 7CC-substituted monomers subjected to polymerization [15,17,18,19,20,21,22,23].

Among the studied cyclic alkylene carbonates, five- and six-membered cycles, including substituted ones, are most common due to their higher stability and availability compared to seven-membered rings. For example, five-membered cyclic carbonates can be prepared from CO_2_ and biomass-derived epoxides [13,14]. Seven-membered cycles (7CCs) are much less common [15], although larger ring-strained carbonates such as 7CCs are more prone to ROP compared to five- or six-membered ones [16]. To the best of our knowledge, only several 7CC-substituted monomers have been subjected to polymerization to prepare and characterize their corresponding polymers (Figure 1), with the substituents being alkyl, alkenyl, phenyl, bis-hydroxy, fused cycloalkyl, cycloalkenyl, heterocycle and naphthalene [15,17,18,19,20,21,22,23].

Some of these copolymers, as well as unsubstituted tetramethylene carbonate (TEMC), were studied with regard to not only their homopolymerization but also their copolymerization with L-Lactide (L-LA) and ε-caprolactone (ε-CL). Of interest, copolymers of L-LA and TEMC demonstrated lower crystallinity and higher enzymatic degradability than the closely related copolymers of L-LA and cyclic carbonates with a shorter alkylene bridge [24,25]. A single (Figure 1) functionally substituted seven-membered carbonate, (5S,6S)-dimethyl-5,6-isopropylidene-1,3-dioxepin-2-one, which is a polymer containing two hydroxy groups in one monomer unit, demonstrated a very low rate of polymerization compared to caprolactone, yielding a block copolymer under one-shot block polymerization conditions [26], probably due to the presence of two fused rings in the monomer. Therefore, it is necessary to continue the search for suitable monomers of seven-membered carbonates that contain a functional group and demonstrate high polymerization rates.

As part to our research program [27,28,29,30] to prepare different biodegradable copolymers that contain functional groups suitable for further modifications, we report herein the synthesis of a novel β-benzyloxy-substituted tetramethylene carbonate (β-BnO-TEMC, **4**) and its homopolymerization and copolymerization with L-LA, ε-CL and trimethylene carbonate (TMC) in the presence of stannous octoate [Sn(Oct)_2_], as well as the characterization of these prepared polymers.

## 2. Materials and Methods

### Experimental Details

All experimental manipulations were performed under a dry, oxygen-free argon atmosphere using standard Schlenk techniques. The solvents were dried using standard methods and distilled prior to use: toluene was refluxed under Na and distilled; diethyl ether was stored under KOH, refluxed under Na/benzophenone and then distilled; methylene chloride was refluxed under CaH_2_ and then distilled; and methanol was refluxed under Mg and then distilled. The starting material, diethyl 2-(benzyloxy) succinate (**2**), was synthesized according to the procedures described in the literature [31]. Diethyl (±) malate (Macklin), benzyl bromide (Macklin) and triphosgene (Macklin) were used as supplied. L-LA was recrystallized from toluene and sublimed in vacuo; trimethylene carbonate was recrystallized from diethyl ether/THF; and ε-Cl was distilled over CaH_2_. ^1^H (400.13 MHz), ^13^C (100.61 MHz) and NMR spectra were recorded using a Bruker Avance 400 or an Agilent 400-MR spectrometer at room temperature (unless otherwise stated). ^1^H and ^13^C chemical shifts were reported in ppm relative to Me_4_Si as the external standard. Gel permeation chromatography (GPC) was carried out on an HPLC chromatograph (column phenogel of 10^4^ Å, refractive index detector); the solvent was THF, the flow rate was 1 mL/min, the sample concentration was 1% and the sample volume was 200 μL. Calibration of the systems were carried out according to polystyrene standards. Differential scanning calorimetry (DSC) was performed using a synchronous thermal analysis instrument, STA 449 F3 Jupiter (Netzsch GmbH & Co., Selb, Bavaria, Germany). High-resolution mass spectra (HRMS) were recorded with a G3 QTof, a quadrupole-time-of-flight mass spectrometer (Waters Corp., Milford, MA, USA) equipped with electrospray ionization (ESI) operated under the positive- and negative-ion modes. The conditions were as follows: an ion source temperature of 550 °C; a drying gas (nitrogen) pressure of 40 psi; a nebulizing gas pressure of 40 psi; a curtain gas pressure of 25 psi; a capillary voltage of 5500 V; and acetonitrile as the solvent.

Diethyl 2-benzyloxysuccinate (2): Following the procedure described in the literature [31], a suspension of diethyl (*±*)-malate **1** (1.72 g, 9.0 mmol) and silver oxide (6.32 g, 27.3 mmol) in 25 mL of EtOAc was added to benzyl bromide (2.16 mL, 18.1 mmol). The mixture was stirred in the dark for two days at rt. The resulting mixture was filtered through Celite and concentrated in vacuo. The residue was purified via column chromatography on silica gel (eluent: EtOAc: PE = 1:10) to yield compound **2** (2.02 g, 79%) as a colorless oil. The R*f* was 0.28. The ^1^H NMR (400 MHz, Chloroform-*d*) spectrum was as follows: δ (ppm) 7.37–7.27 (m, 5H, aromatic protons), 4.78 (d, *J* = 11.4 Hz, 1H, PhC*H*_2_), 4.54 (d, *J* = 11.4 Hz, 1H, PhC*H*_2_), 4.39 (dd, *J* = 7.8, 5.1 Hz, 1H, C*H*OBn), 4.25-4.11 (m, 4H, CH_3_C*H*_2_O), 2.86–2.67 (m, 2H, OCHC*H*_2_CO), 1.29, 1.25 (2t, *J* = 7.1 Hz, 6H, C*H*_3_CH_2_O). The ^13^C NMR (101 MHz, CDCl_3_) spectrum was as follows: δ (ppm) 171.47 (*C*O), 170.16 (*C*O), 137.40, 128.46, 128.19, 128.02 (aromatic carbons), 74.77 (*C*HOCH_2_Ph), 73.15 (*C*H_2_Ph), 61.33 (CH_3_*C*H_2_O), 60.96 (CH_3_CH_2_O), 38.17 (*C*H_2_CHOBn), 14.27 (*C*H_3_CH_2_O), 14.22 (*C*H_3_CH_2_O).

2-(Benzyloxy)butane-1,4-diol (3): An Et_2_O solution (25 mL) of diester **2** (2.50 g, 8.9 mmol) was added dropwise to a suspension of LiAlH_4_ (0.80 g, 21 mmol) in Et_2_O (20 mL) under an argon atmosphere at 0 °C. The gray suspension was vigorously stirred for 12 h. The mixture was cooled to 0 °C and quenched via a successive dropwise addition of H_2_O (2.4 mL), a 15% NaOH solution (2.4 mL) and H_2_O (4.8 mL). After dilution with EtOAc (40 mL), it was filtered and dried over Na_2_SO_4_. Then, the organic solvent was removed under reduced pressure. Compound **3** (1.57 g, 90%) was isolated as a colorless oil. The ^1^H NMR (400 MHz, Chloroform-*d*) spectrum was as follows: δ (ppm) 7.37–7.29 (m, 5H, aromatic protons), 4.78 (d, *J* = 11.4 Hz, 1H, PhCH_2_), 4.61-4.59 (m, 2H, PhCH_2_), 3.8–3.68 (m, 4H, CH_2_OH), 3.62-3.58 (m, 1H, CHOBn), 2.59 (br. s, 2H, OH), 1.88–1.80 (m, 2H, OCHCH_2_CH_2_). The ^13^C NMR (101 MHz, CDCl_3_) spectrum was as follows: δ (ppm) 138.22, 128.67, 128.04, 127.97 (aromatic carbons), 77.90 (CHOCH_2_Ph), 71.75 (CH_2_Ph), 64.04 (HOCH_2_CHOBn), 59.57 (HOCH_2_CH_2_), 34.00 (HOCH_2_CH_2_).

5-(benzyloxy)-1,3-dioxepan-2-one (4): A solution of triphosgene (0.85 g, 2.9 mmol) in dichloromethane (85 mL) was added dropwise over 15 min to a stirred solution of diol **3** (1.12 g, 5.7 mmol) and anhydrous pyridine (2.76 mL, 2.71 g, 34.2 mmol) in dichloromethane (280 mL) at −78 °C. After 75 min at −78 °C, the reaction was quenched via the addition of a saturated aqueous solution of ammonium chloride (80 mL) followed by water (20 mL). The mixture was allowed to warm to 5 °C, and the organic phase was separated. The aqueous phase was extracted with dichloromethane (3 × 50 mL). Then, the organic extracts were combined, washed with brine (100 mL), dried over Na_2_SO_4_ and concentrated. The residue was purified via column chromatography on silica gel (eluent: EtOAc:PE = 1:1) to yield compound **4** at R*f* = 0.80 (0.69 g, 55%) as a colorless oil and compound **5** at R*f* = 0.25 (0.06 g, 6%) as a yellowish oil. For compound **4**, the ^1^H NMR (400 MHz, Chloroform-*d*) spectrum was as follows: δ (ppm) 7.37–7.31 (m, 5H, aromatic protons), 4.60 (dd, *J* = 23.2, 11.4 Hz, 2H, PhC*H*_2_), 4.50–4.44, 4.28–4.23, 4.19–4.09 (3m, 4H, C*H*_2_O), 3.78–3.75 (m, 1H, C*H*OBn), 2.12–2.09 (m, 2H, OCHC*H*_2_CH_2_). The ^13^C NMR (101 MHz, CDCl_3_) spectrum was as follows: δ (ppm) 154.73 (*C*O), 137.53, 128.68, 128.15, 127.76 (aromatic carbons), 72.02 (*C*HOCH_2_Ph), 70.86 (O*C*H_2_CHOBn), 70.41 (*C*H_2_Ph), 66.19 (O*C*H_2_CH_2_) and 33.13 (OCH_2_*C*H_2_). HRMS (ESI) was calculated for C_12_H_14_O_4_ [M+H]^+^, with *m/z* 223.0965 corresponding to [M+H]^+^, and *m/z* 223.0963.

For compound **5** (3-benzyloxytetrahydrofuran), the ^1^H NMR (400 MHz, Chloroform-*d*) spectrum was as follows: δ (ppm) 7.37–7.26 (m, 5H, aromatic protons), 4.64–4.55 (m, 2H, PhCH_2_), 4.25–4.13 (m, 2H, CH_2_O), 3.8–3.69 (m, 2H, CH_2_O), 2.14–2.03 (m, 1H, OCH_2_CH_2_CH) and 2.95–1.84 (m, 1H, OCH_2_CH_2_CH).

Typical Polymerization Procedure in Solution: Sn(Oct)_2_ in toluene was added to the solution of the monomer in toluene. The reaction mixture was heated at 100 °C. The degree of conversion was determined via ^1^H NMR spectroscopy. The reaction was terminated through the addition of MeOH. The solvents were then removed in vacuo. Methylene chloride was added to the dry residue until complete dissolution. The resulting solution was poured into methanol with vigorous stirring. The precipitated polymer was separated and dried in vacuo.

## 3. Results and Discussion

### 3.1. Preparation of β-BnO-TEMC, ***4***

**Scheme 1:**
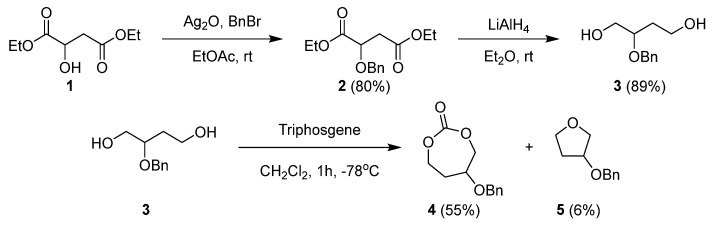
Preparation of carbonate **4**.

The novel carbonate **4** was synthesized according to Scheme 1 from previously prepared diol via the cyclization of racemic diol **3** [31], with 0.5 equiv. of triphosgene and 6 equiv. of pyridine in CH_2_Cl_2_ at –78 °C. The residue was purified via column chromatography on silica gel (the eluent was as follows: EtOAc:PE, 1: 1; R_f_(**4**) = 0.7 and R_f_(**5**) = 0.2) to yield the novel carbonate **4** (55%) as a colorless oil and **5** (6%) as a yellowish oil. The fact that carbonate **4** was isolated by means of column chromatography demonstrates the sufficient stability of the resulting carbonate. The by-product tetrahydrofuran **5** can be formed at the stage of acylation of alcohol **3** with triphosgene, as this reaction can then proceed in two different ways: in the case of 7-*exo*-trig cyclization, the desired carbonate **4** is obtained, while the closure of the 5-*exo*-tet ring via an S_N_2 reaction leads to the formation of tetrahydrofuran **5**. The formation of a similar by-product has also been observed previously [32]. Maintaining the temperature regime of this reaction is extremely important. When the reaction is carried out at room temperature, compound **5** (3-benzyloxy-tetrahydrofuran) can be produced in a sufficient amount. Diol **3** was prepared from commercially available diethyl (±)-malate in two steps: O-benzylation of **1** by BnBr in the presence of Ag_2_O in EtOAc at r.t. and then a lithium aluminum hydride reduction of **2** in Et_2_O for 24 h. at r.t. (Scheme 1) [31].

### 3.2. Ring-Opening Polymerization of 4 (Homopolymerization and Copolymerization)

**Scheme 2:**
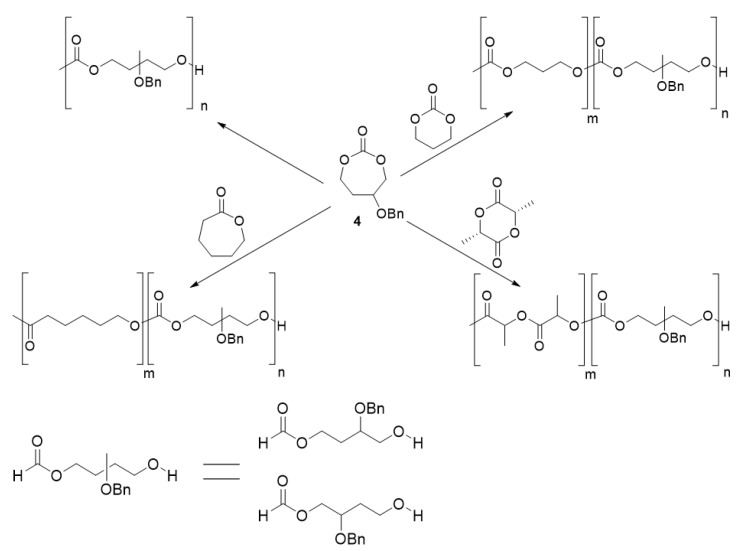
Polymerization of carbonate **4** and its copolymerization with L-LA, ε-CL and TMC.

Sn(Oct)_2_ was chosen as a catalyst for the ring-opening homopolymerization of the monomer (β-BnO-TEMC, **4**) as well as for the copolymerization of monomer **4** with L-LA, ε-CL and TMC as comonomers under different ratios of the monomer. Sn(Oct)_2_ is the most often used catalyst/initiator system for the polymerization of lactones and cyclic carbonates [33]. The polymerization processes were investigated in toluene at 100 °C (Scheme 2). The monomer-to-catalyst ratio (M/C) was 55:1 for the sum of the monomers in each reaction. Sn(Oct)_2_ was used by us without alcohols as co-initiators in a series of polymerization experiments. Usually, residual water is considered a co-initiator in this type of polymerization system [26].

**Figure 2:**
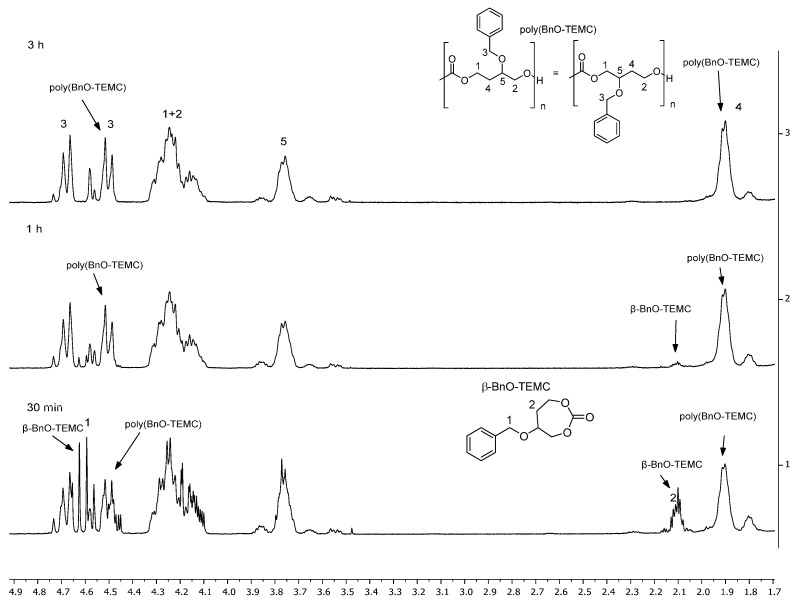
^1^H NMR (CDCl_3_) spectra for Sn(Oct)_2_-catalyzed polymerization of β-BnO-TEMC (**4**) under various reaction times (Table 1, Entry 1).

The results of the homopolymerization of **4** are presented in Table 1 (Entry 1). The polymerization rate was expected to be high: a 93% conversion in 1 h under the conditions described above (Figure 2). It should be noted that both the previously obtained substituted 7CC and unsubstituted TEMC polymerize under harsher conditions (usually in bulk at 120 °C) [26].

The regioregularity of poly(β-BnO-TEMC) was investigated using ^13^C{^1^H} NMR spectroscopy. In the carbonyl region (δ 155.0, 155.1 and 155.2 ppm) of the spectrum of poly(β-BnO-TEMC), we observed three resonances with a relative intensity ratio of 1:2:1 (see Supplementary Materials, Figure S9). These data indicate that the ROP of poly(β-BnO-TEMC) under the action of Sn(Oct)_2_ occurs in a statistical regime (the remote position of the benzyl substituent in β-BnO-TEMC does not allow the initiator to distinguish between the two O−C(O)O bonds), which is generally consistent with the results obtained for other β-substituted 7CCs [15].

Additionally, homopolymerization with ε-CL, L-LA and TMC was carried out under the conditions described above (Table 1, Entries 2, 6 and 8). The polymerization rate was lower than that for **4**: conversion in 6 h (L-LA—77%, ε-CL—89% and TMC—50%). It should be noted that the polymerization rate of the seven-membered cycle (ε-CL) was also expected to be higher than the polymerization rate of the six-membered cycles (L-LA and TMC). The molecular weight distribution of poly(**4**) as determined by GPC was significantly narrow (*Ð* = 1.26). These data illustrate a good degree of control over molecular weights. The small molecular weight of poly(**4**) can be explained by the fairly frequent breakage of the polymerization chain, which is often found in substituted cyclic esters [34].

The results of copolymerization of **4** with L-LA, ε-CL and TMC are presented in Table 1 (Entries 3–5, 7 and 9). We studied the copolymerization of **4** with ε-CL at three different monomer ratios: 1:10, 1:5 and 1:1. We studied the copolymerization with L-LA and TMC at only the ratio of 1:1 in the experiments. The molecular weights of the 1:1 copolymers are noticeably less than the molecular weights of the homopolymers (L-LA, ε-CL and TMC); at the same time, copolymerization of ε-CL and **4** at a ratio of 10:1 yields a polymer with a large mass (for a given amount of the monomer). The resulting copolymer (Entry 3) can be considered a promising polymer after the deprotection of the hydroxy group for the inoculation of the functional substituents.

It is important to note that the polymerization rate of the three “classical” unsubstituted monomers used increases under copolymerization with compound **4** compared with the corresponding homopolymerization. Thus, compound **4** is a promoter of L-LA and ε-CL copolymerization. At the same time, TMC polymerizes with **4** at approximately the same rate under both homopolymerization and copolymerization conditions.

The copolymers were also characterized using ^1^H and ^13^C NMR spectroscopy in CDCl_3_. The comonomer sequence distributions were determined from the spectral data (Table 2). In the ^1^H NMR spectrum of poly(OBn-TEMC-*co*-LA) (Table 1, Entry 7), the signals at 5.02 and 1.52 ppm were assigned to the corresponding methine and methyl protons of the TEMC-LA heterosequences (see Supplementary Materials, Figure S12). In addition, in the ^1^H NMR spectrum of the poly(OBn-TEMC-*co*-LA), we observed a peak at 5.17 ppm corresponding to the LA–LA diad. In the ^13^C NMR spectrum of poly(OBn-TEMC-*co*-LA) (Table 1, Entry 7), the peaks at 155.11–155.21 and 154.43–154.57 ppm correspond to TT and TL diads, respectively. For poly(OBn-TEMC-*co*-CL) (Table 1, Entry 5), the peaks at 173.87, 173.69, 173.43 and 173.39 were found in the carbonyl region of the ^13^C NMR spectrum, which correspond to the triads CL-CL-CL, TEMC-CL-CL, CL-CL-TEMC and TEMC-CL-CL, respectively, while the TEMC-CL and TEMC-TEMC diads correspond to the peaks at 155.12 and 155.23–155.33 ppm, respectively. These assignments are in accordance with the literature data [27,35,36,37]. Using the ^1^H and ^13^C NMR spectroscopy data, the average sequence lengths *L*_TEMC_ and *L*_M_ (where M = ε-CL and L-LA) were estimated according to Equations (S1)–(S4) in the Supplementary Materials. During the copolymerization process initiated by Sn(Oct)_2_, the consumption of **4** was completed faster than that for L-LA and ε-CL; thus, random copolymers were formed through transesterification. The ^13^C NMR spectra of poly(TEMC-*co*-TMC) (Table 1, Entry 9), poly(TEMC -*co*-CL) (Table 1, Entry 3) and poly(TEMC -*co*-CL) (Table 1, Entry 3) do not contain the resonances expected of diad and triad sequences originating from a random copolymer, suggesting the formation of gradient copolymers. All the obtained copolymers presented in Table 1 had a unimodal molecular weight distribution (see Supplementary Materials, Figure S24), with a polydispersity index ranging from 1.26 to 1.47, which indicates that the obtained polymers are pure copolymers without homopolymers of L-LA, ε-CL, TMC and **4**.

**Figure 3:**
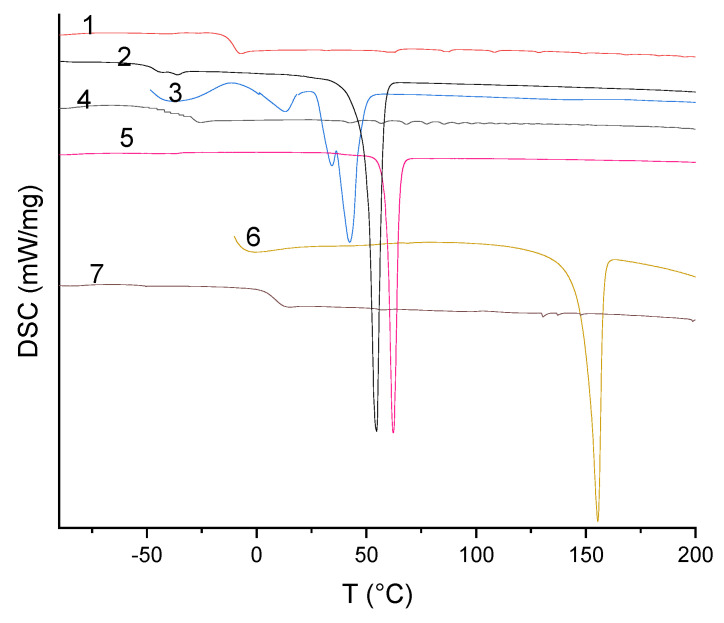
DSC thermograms for the poly(BnO-TEMC) homopolymer (1) and its copolymers with ε-CL and L-LA: (2) at a ratio of 10:1 for (ε-CL):(**4**), (3) at a ratio of 5:1 for (ε-CL):(**4**), (4) at a ratio of 1:1 for (ε-CL):(**4**), (5) poly(ε-CL), (6) poly-L-LA, and (7) at a ratio of 1:1 for (L-LA):(**4**). Scanning using a DSC 204 F1 (Netzsch, Germany) at a heating rate 10 °C/min in the temperature range from −100 to 200 °C under an inert atmosphere with an argon flow rate 60 mL/min in aluminum crucibles.

Figure 3 shows the DSC thermograms for the poly(BnO-TEMC) homopolymer, its copolymers with L-LA and ε-CL, and the homopolymers of L-LA and ε-CL synthesized and isolated under the same conditions. It is evident that the poly(BnO-TEMC) homopolymer has a glass transition temperature of −10 °C (Figure 3, line 1). The ε-CL homopolymer has high crystallinity and exhibits a melting temperature of 60 °C (Figure 3, line 5). When copolymerizing with β-BnO-TEMC in excess of ε-CL, the length of the ε-Cl blocks in the chain decreases with an increase in the β-BnO-TEMC content, which leads to a decrease in the melting temperature to 54 °C at a monomer ratio of 10:1 and to 42.9 °C at a monomer ratio of 5:1 (Figure 3, lines 2 and 3, respectively). Further increase in the content of the β-BnO-TEMC comonomer to an equivalent composition leads to the complete disappearance of the melting peak, which indicates a statistical distribution of the links in the copolymer chain (Figure 3, line 4).

A similar tendency was observed for the β-BnO-TEMC and L-LA copolymer of equivalent composition. The copolymer does not exhibit crystallinity and demonstrates one glass transition temperature of +8.5 °C (Figure 3, line 7).

## 4. Conclusions

In the course of this work, a series of copolymers containing a previously unknown functionally substituted β-BnO-TEMC as one of the monomers were obtained. The use of this carbonate in copolymerization accelerates the polymerization rate of L-LA and ε-CL compared with the data obtained under the same conditions (solution in toluene at 100 °C, with Sn(Oct)_2_ as the catalyst) with respect to the rates of their homopolymerization. The polymerization rate of TMC changes little. All the compounds obtained are copolymers. The copolymers with a 1:1 composition have a relatively low molecular weight, do not crystallize and show a T_g_ corresponding to a distribution of units that is close to a statistical distribution. The copolymers with an excess of caprolactone crystallize, and those with a higher caprolactone content in the chain were found to have a higher T_m_. The copolymer with a composition of 10:1 of (ε-CL):(**4**) can be considered a promising polymer after the deprotection of the hydroxy group for the inoculation of the functional substituents due to its convenience of preparation and properties similar to those of poly(ε-caprolactone).

## Figures and Tables

**Table 1:** Results of homopolymerization of **4**, ε-CL, L-LA, TMC and their copolymerizations catalyzed by Sn(Oct)_2_ ^1^.

Entry	Monomer 1	Monomer 2	[M1]:[M2]: [Sn(Oct)_2_]	Time, h	Conversion ^2^ [%]	M_n_^3^ [g/mol]	*Ð* ^3^
1	**4**	-	55:1	0.5	72	-	-
				1	93	-	-
				3	100	2674	1.26
2	ε-CL	-	55:1	1	10	-	-
				3	37	-	-
				6	89	-	-
				24	92	8679	1.34
3	**4**	ε-CL	5:50:1	48		9600	1.47
4	**4**	ε-CL	9.16:45.84:1	48		5229	1.38
5	**4**	ε-CL	27.5:27.5:1	1	100; 63	-	-
				3	100; 76	-	-
				6	100; 90	-	-
				24	100; 96	3638	1.26
6	L-LA	-	55:1	1	20	-	-
				3	50	-	-
				6	77	-	-
				24	93	6488	1.28
7	**4**	L-LA	27.5:27.5:1	1	100; 76	-	-
				3	100; 93	-	-
				6	100; 95	-	-
				24	100; 95	3789	1.36
8	TMC	-	55:1	1	6	-	-
				3	25	-	-
				6	48	-	-
				24	96	4463	2.73
9	**4**	TMC	27.5:27.5:1	1	100; 14	-	-
				3	100; 20	-	-
				6	100; 50	-	-
				24	100; 86	3256	1.30

^1^ Conditions: Polymerization in toluene solution, [monomer] = 0.5 M, 100 °C; ^2^ Determined using ^1^H NMR; ^3^ Determined using GPC vs. polystyrene standards (uncorrected raw data).

**Table 2:** The relative contents of each monomer and the average sequence lengths of the (M)*_n_* and (TEMC)*_m_* fragments.

Entry	M	[TEMC]:[M]	*L*_TEMC_	*L*_M_	TEMC:M in Obtained Polymer ^1^, %
1	ε-CL	1:10	-	-	8:92
2	ε-CL	1:5	-	-	17:83
3	ε-CL	1:1	1.7	2.2	55:45
4	L-LA	1:1	1.7	2.6	51:49
5	TMC	1:1	-	-	67:33

^1^ The relative contents of each monomer as determined by ^1^H NMR spectroscopy in CDCl_3_.

## Data Availability

Data is contained within the article or Supplementary Material.

## Supplementary Materials

The following supporting information can be downloaded at https://www.mdpi.com/article/10.3390/polym16233364/s1: additional NMR spectra of monomer **4** and polymers, equations for calculating the average sequence lengths of L-LA, ε-CL, TMC and BnO-TEMC, normalized GPC curves of polymers.
