# Supplementary material for: Novel β-Benzyloxy-Substituted Copolymers of Seven-Membered Cyclic Carbonate: Ring-Opening Polymerization with L-Lactide, ε-Caprolactone and Trimethylenecarbonate
Source: Polymers (Basel). 2024 Nov 29;16(23):3364. doi: 10.3390/polym16233364 (PMC11644486; doi:10.3390/polym16233364)
Supplement: Supplementary file 1 [file polymers-16-03364-s001.zip › polymers-3318334-supplementary.pdf]

Copolymers of Novel β-Benzyloxy-substituted of Seven-Membered Cyclic Carbonate: Ring-Opening Polymerization with L-Lactide, ε-Caprolactone and Trimethylenecarbonate

Valeriia A. Serova [1], Badma N. Mankaev [1], Milana U. Agaeva [1], Elena V. Chernikova [1], Anna K. Berkovich [1], Roman S. Alekseyev [1], Aleksei V. Khvostov [1], Sergey V. Timofeev and Sergey S. Karlov [1]*

[1]Department of Chemistry, Moscow State University, Leninskye Gory 1, Moscow 119991, Russia

Table of contents

1. NMR spectra of compounds **2-4**..S3

2. Equations for calculating the average sequence lengths of L-LA, ε-CL, TMC and BnO-TEMC..S6

3. NMR spectra of polymers...S7

4. Normalized GPC curves of polymers..S16

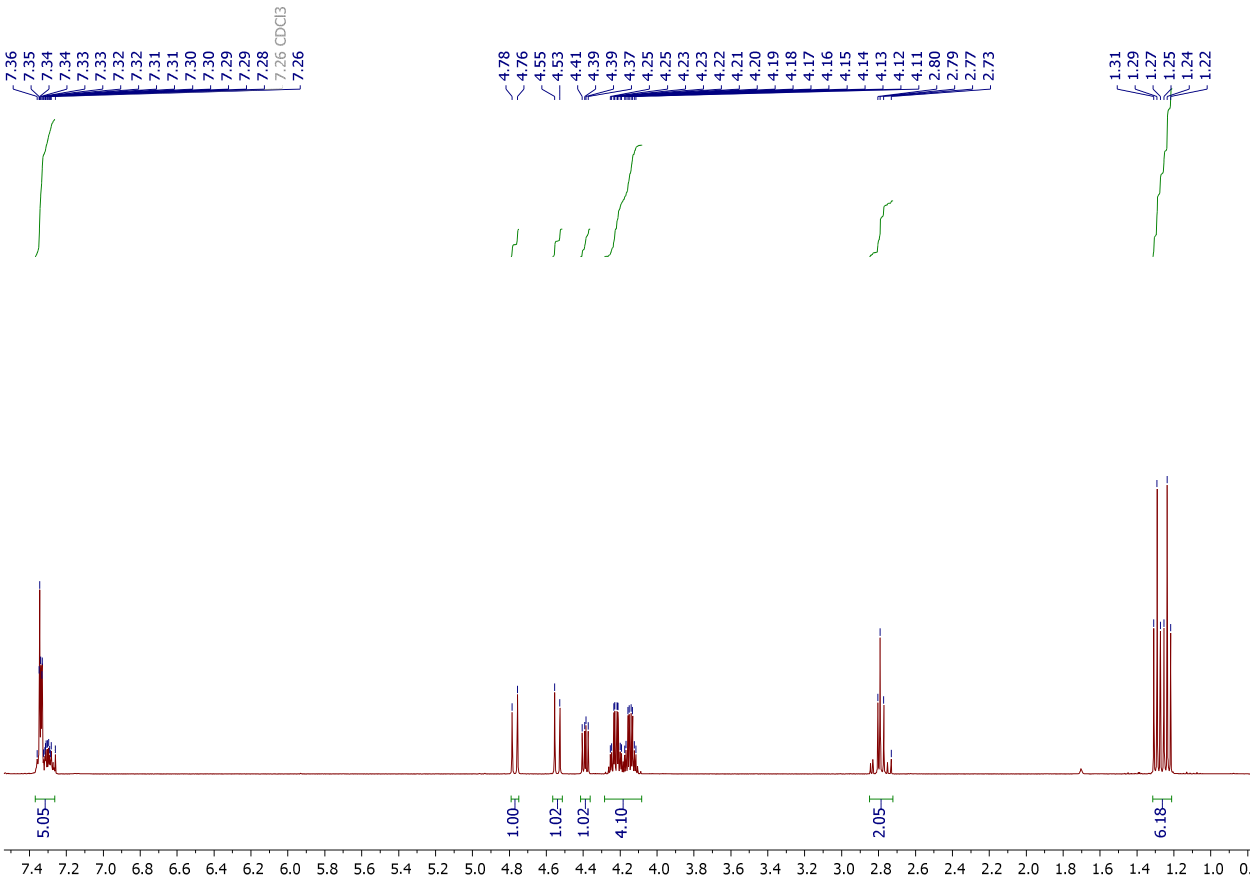

Figure S1. ^{1}H NMR spectrum in $CDCl_3$ of compound **2**

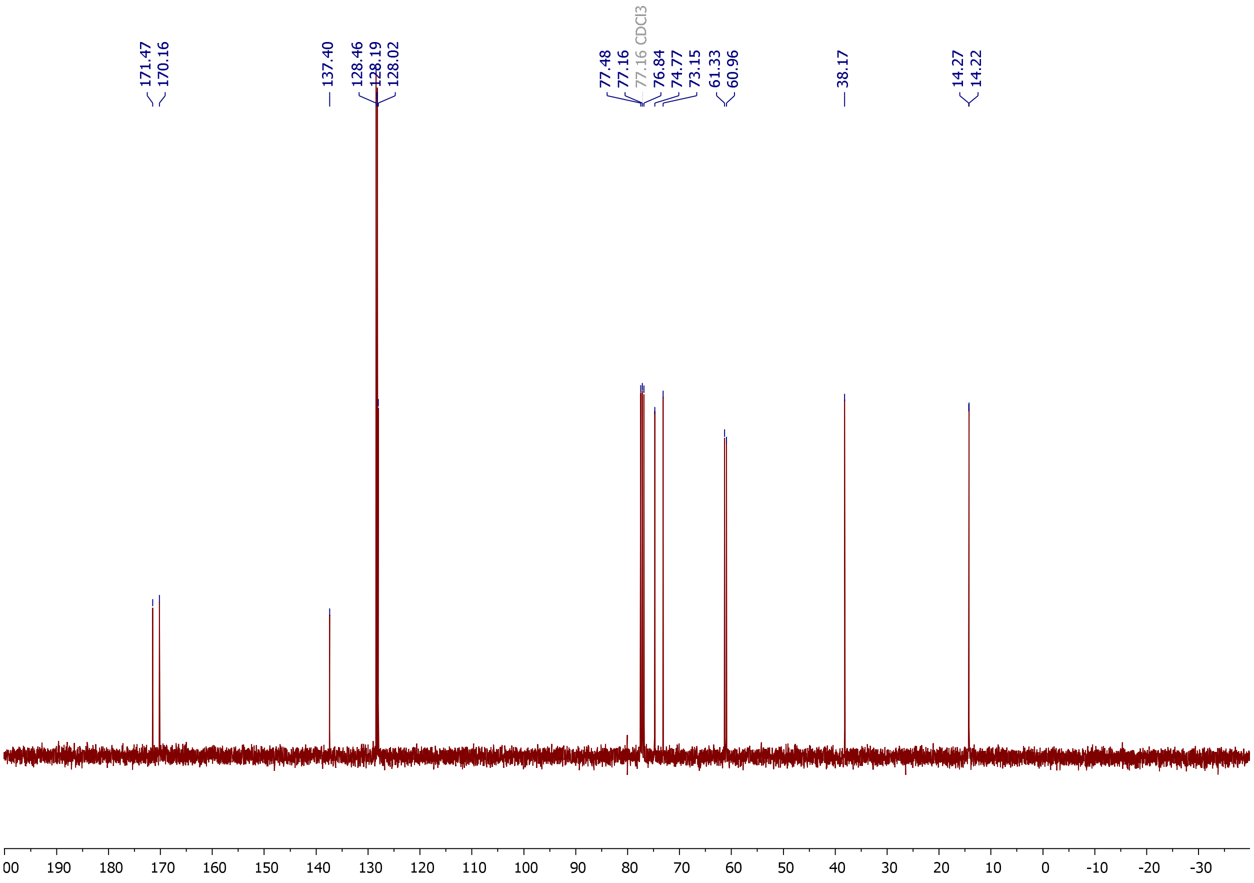

Figure S2. ^{13}C NMR spectrum in $CDCl_3$ of compound **2**

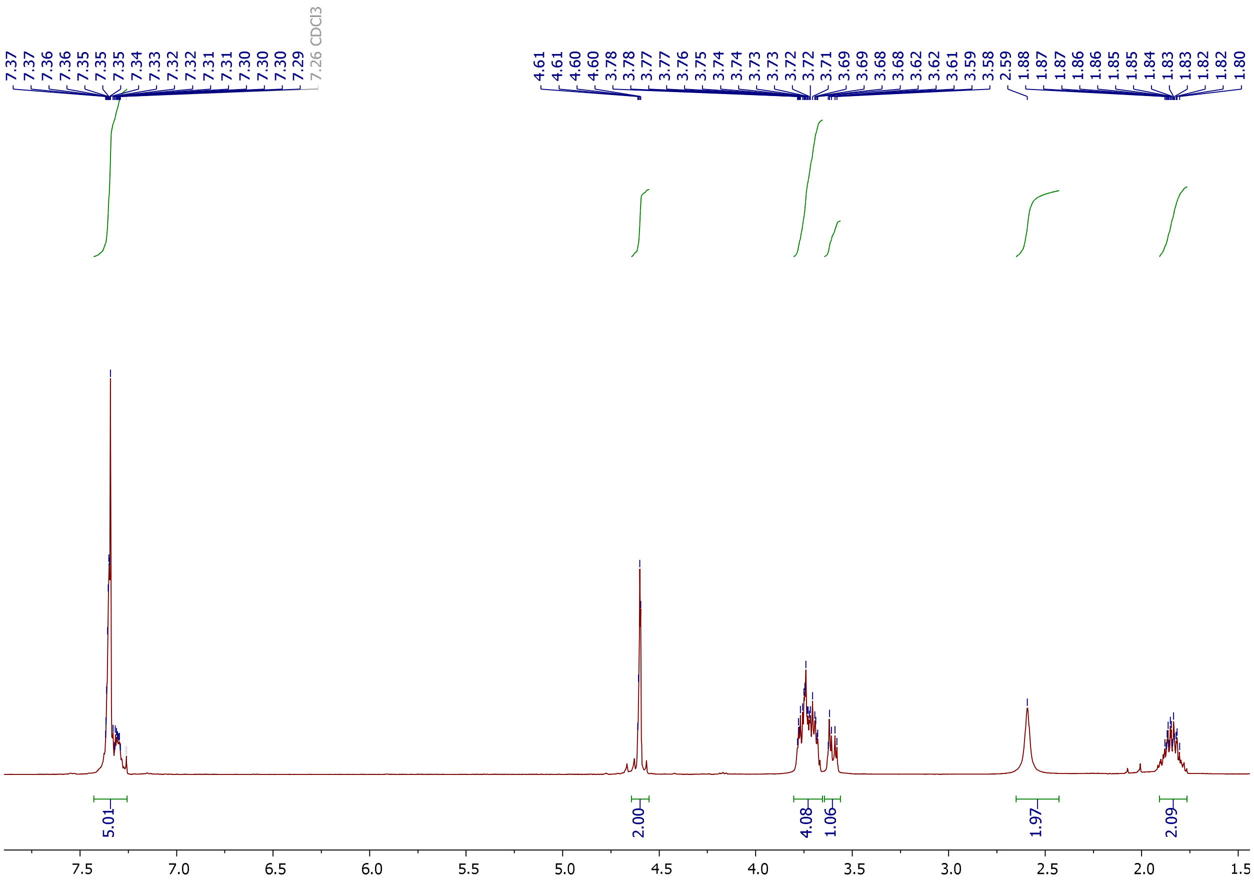

Figure S3. ^{1}H NMR spectrum in $CDCl_3$ of compound **3**

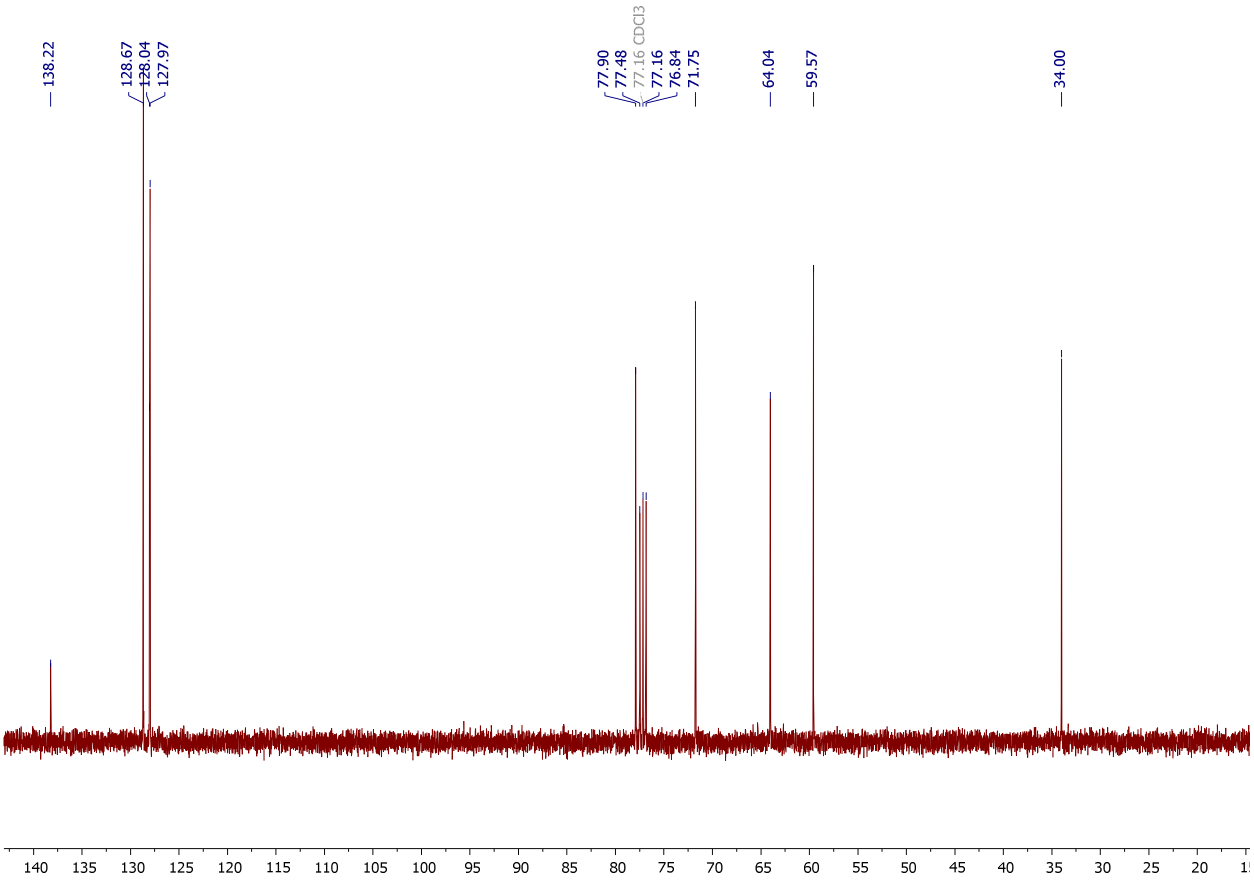

Figure S4. ^{13}C NMR spectrum in $CDCl_3$ of compound **3**

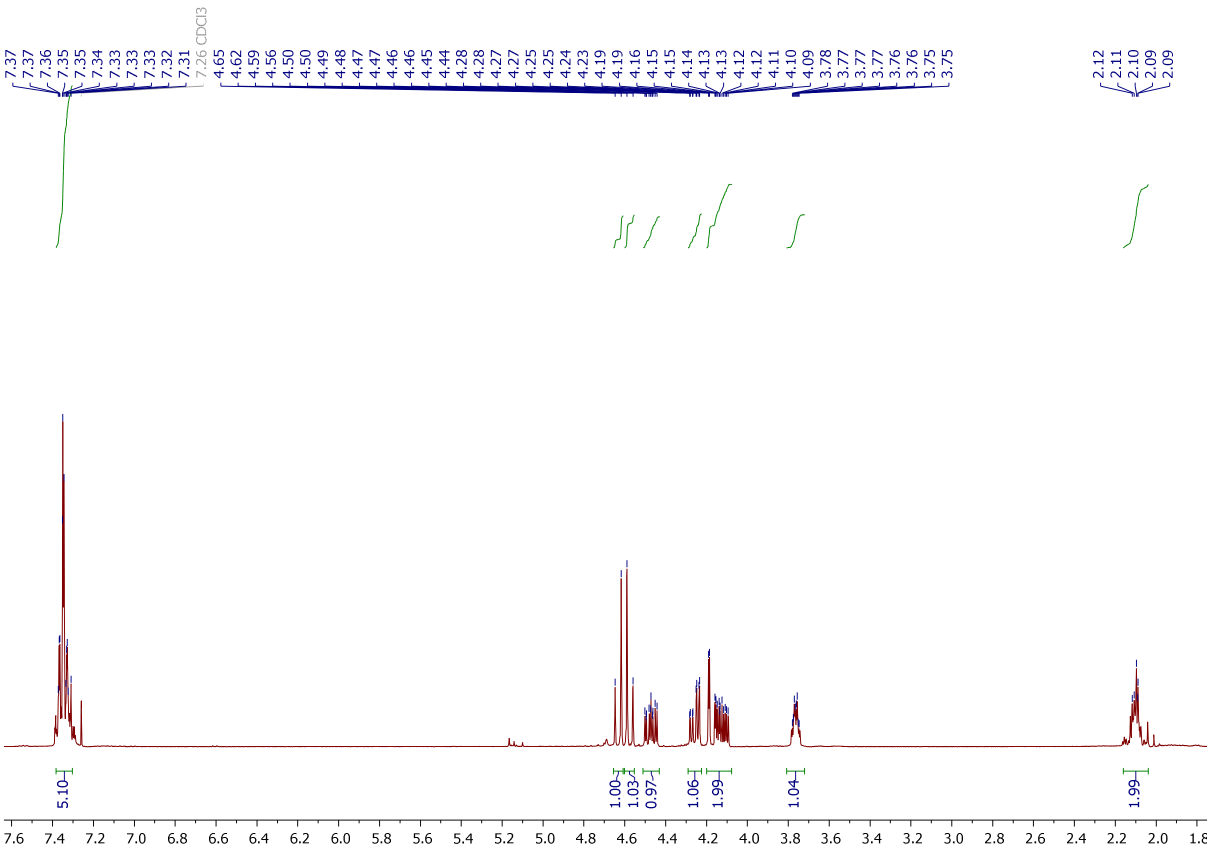

Figure S5. ^{1}H NMR spectrum in $CDCl_3$ of compound β-benzyloxy-substituted tetramethylene carbonate **4**

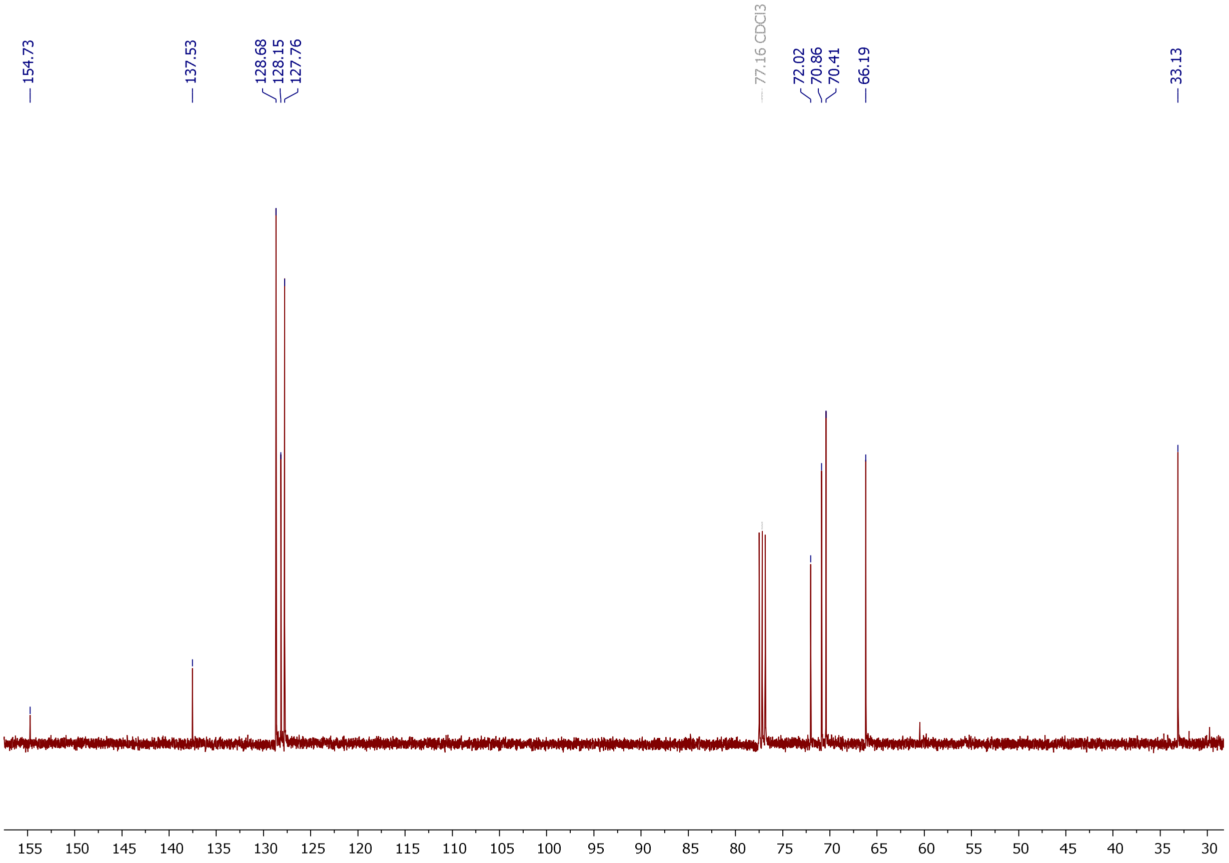

Figure S6. ^{1}H NMR spectrum in $CDCl_3$ of compound β-benzyloxy-substituted tetramethylene carbonate **4**

Calculation of the average sequence length of poly(BnO-TEMC-*co*-LA) [21, 29-31]:

The sequence lengths of L-LA units were obtained from the relative peak intensities at 5.17 ppm (LA–LA diads) and at 5.04 ppm (TEMC–LA diads) (Figure S18) using the following equation: $L_{\mathrm{LA}}=\frac{I_{5.17ppm}}{I_{5.04ppm}}+1$ **(S1)**.

The sequence lengths of TEMC units were calculated from ^{13}C NMR (Figure S19) according to equation **S2**:

$$L_{\mathrm{TEMC}}=\frac{I_{155.11}+I_{155.21ppm}}{I_{154.43}+I_{154.57ppm}}+1 \quad \textbf{(S2)}$$

Calculation of the average sequence length of poly(BnO-TEMC-*co*-CL):

The sequence lengths of CL units were calculated from ^{13}C NMR (Figure S15) according to equation **S3**:

$$L_{\mathrm{CL}}=\frac{I_{173.69}+I_{173.87ppm}}{I_{154.39}+I_{173.43ppm}}+1 \quad \textbf{(S3)}$$

The sequence lengths of TEMC units were calculated from ^{13}C NMR (Figure S15) according to equation **S4**:

$$L_{\mathrm{TEMC}}=\frac{I_{155.23}+I_{155.33ppm}}{I_{155.12ppm}}+1 \quad \textbf{(S4)}$$

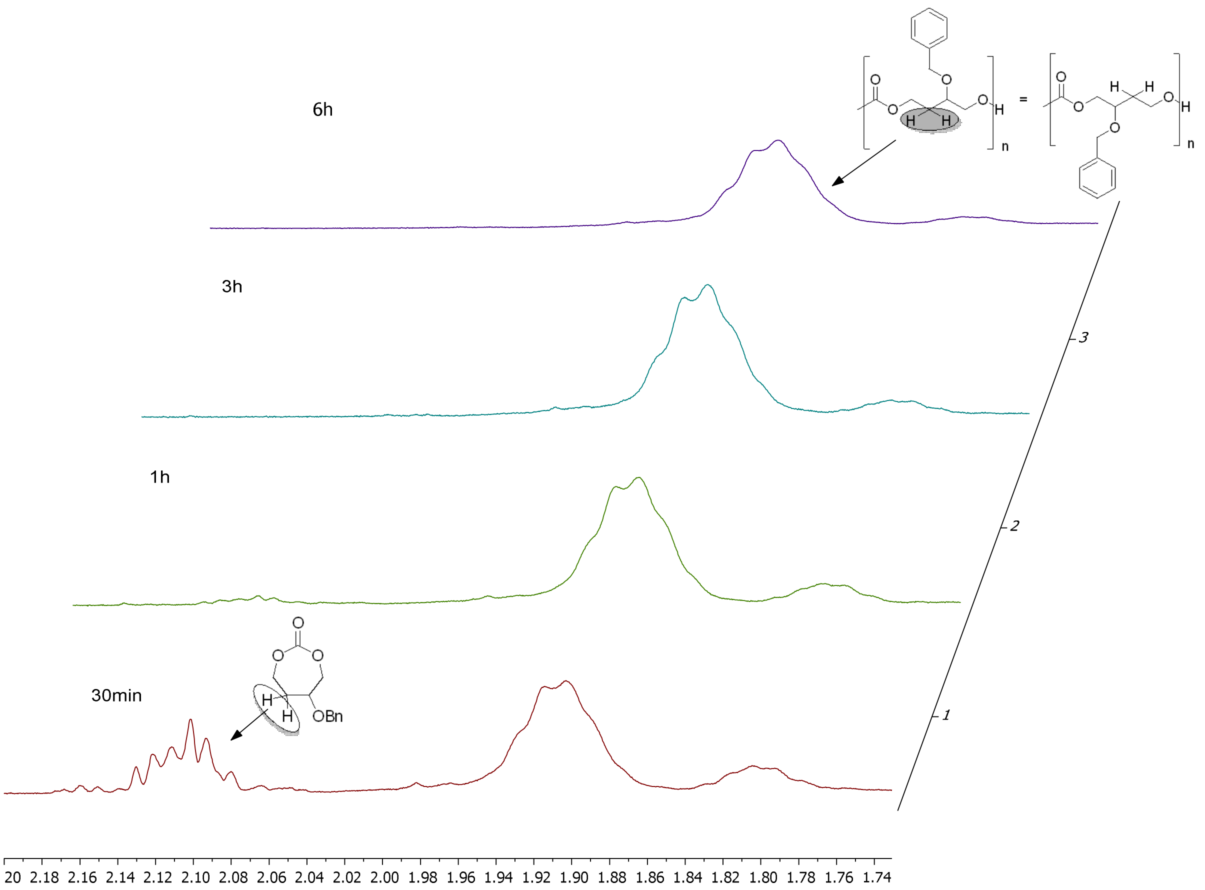

Figure S7. ^{1}H NMR monitoring of β-BnO-TEMC polymerization conversion (Table 1, entry 1).

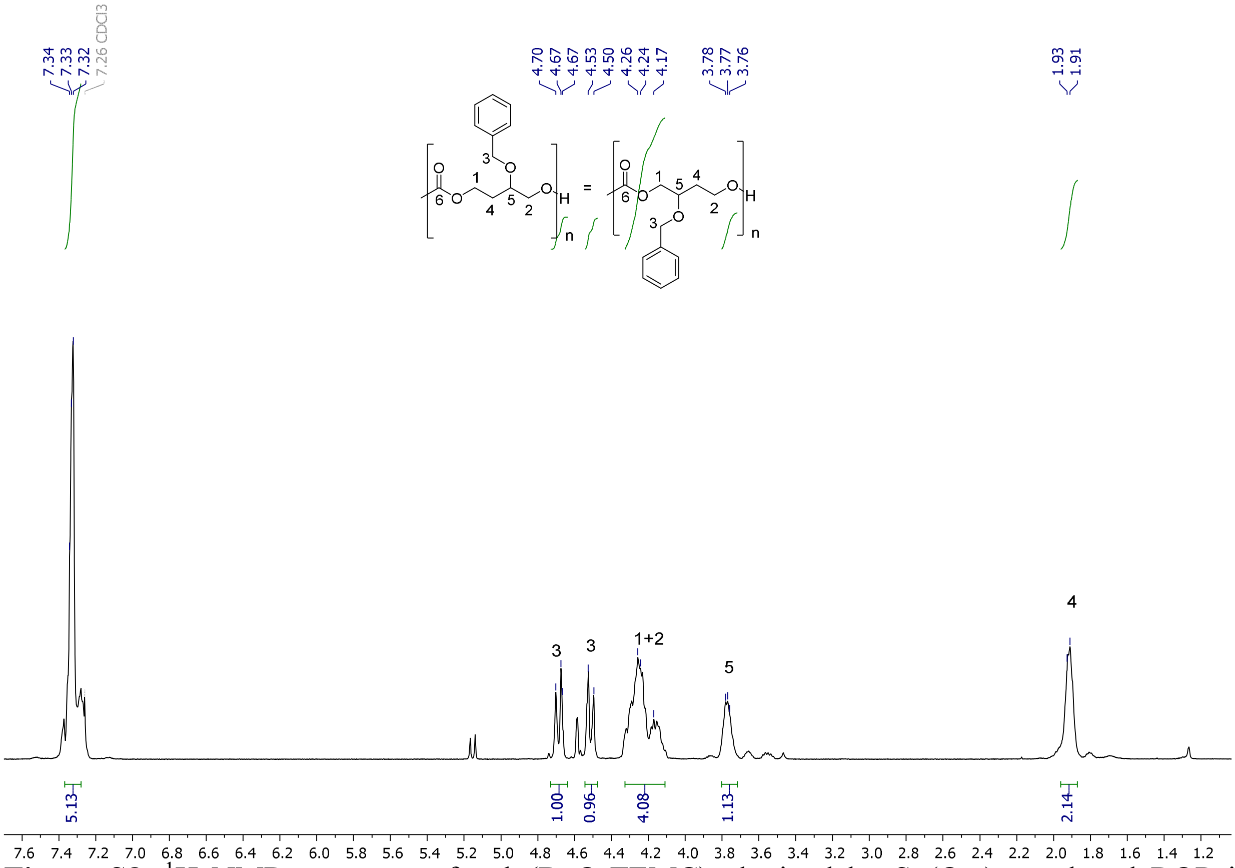

Figure S8. ^{1}H NMR spectrum of poly(BnO-TEMC) obtained by $Sn(Oct)_2$-catalyzed ROP in toluene solution at 100 °C for 6 h (Table 1, entry 1).

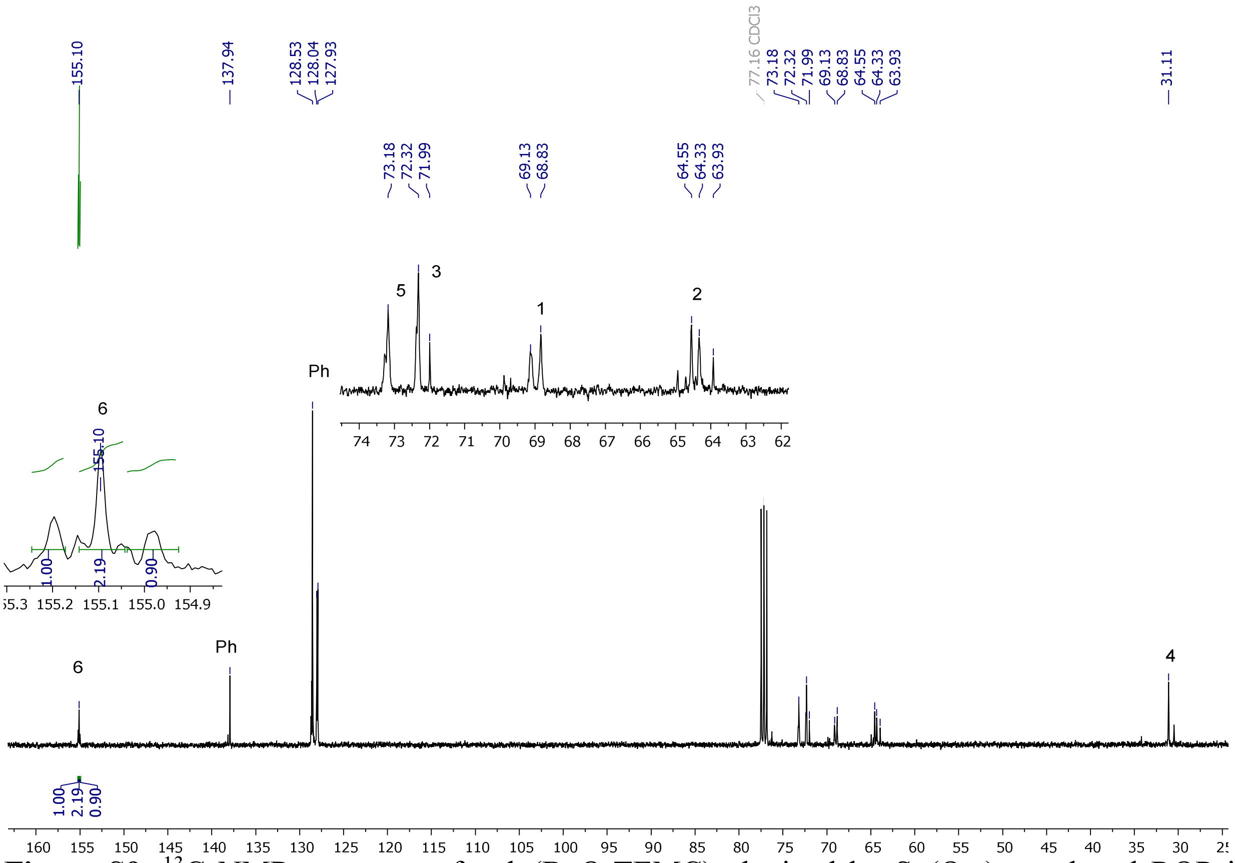

Figure S9. ^{13}C NMR spectrum of poly(BnO-TEMC) obtained by $Sn(Oct)_2$-catalyzed ROP in toluene solution at 100 °C for 6 h (Table 1, entry 1).

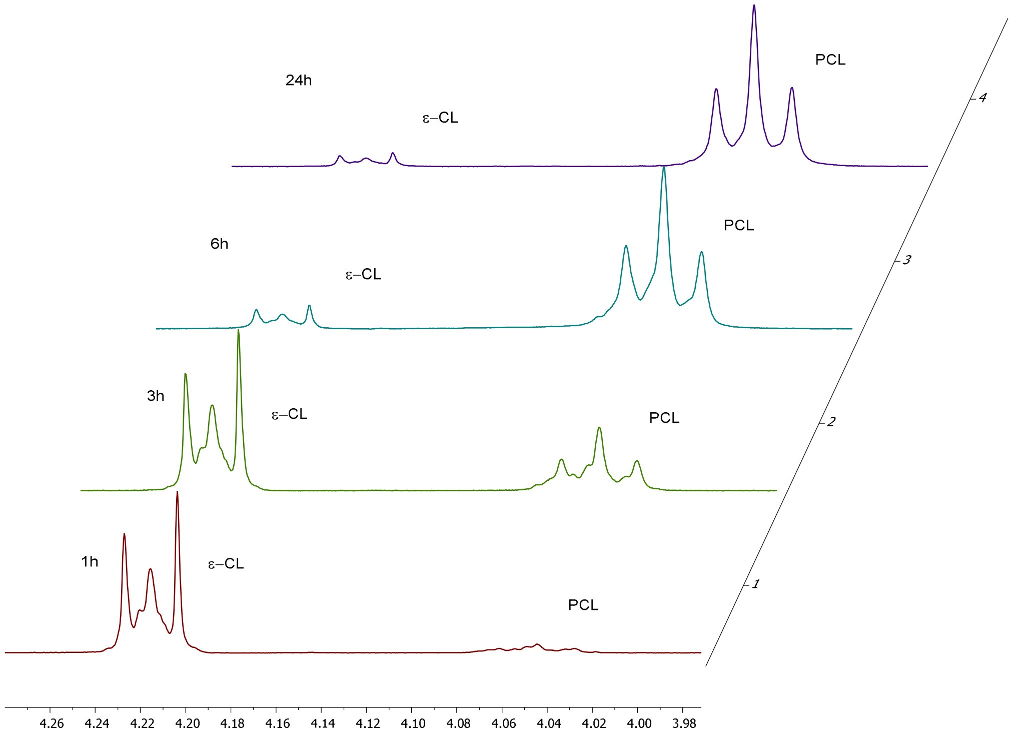

Figure S10. ^{1}H NMR monitoring of ε-caprolactone polymerization conversion (Table 1, entry 2).

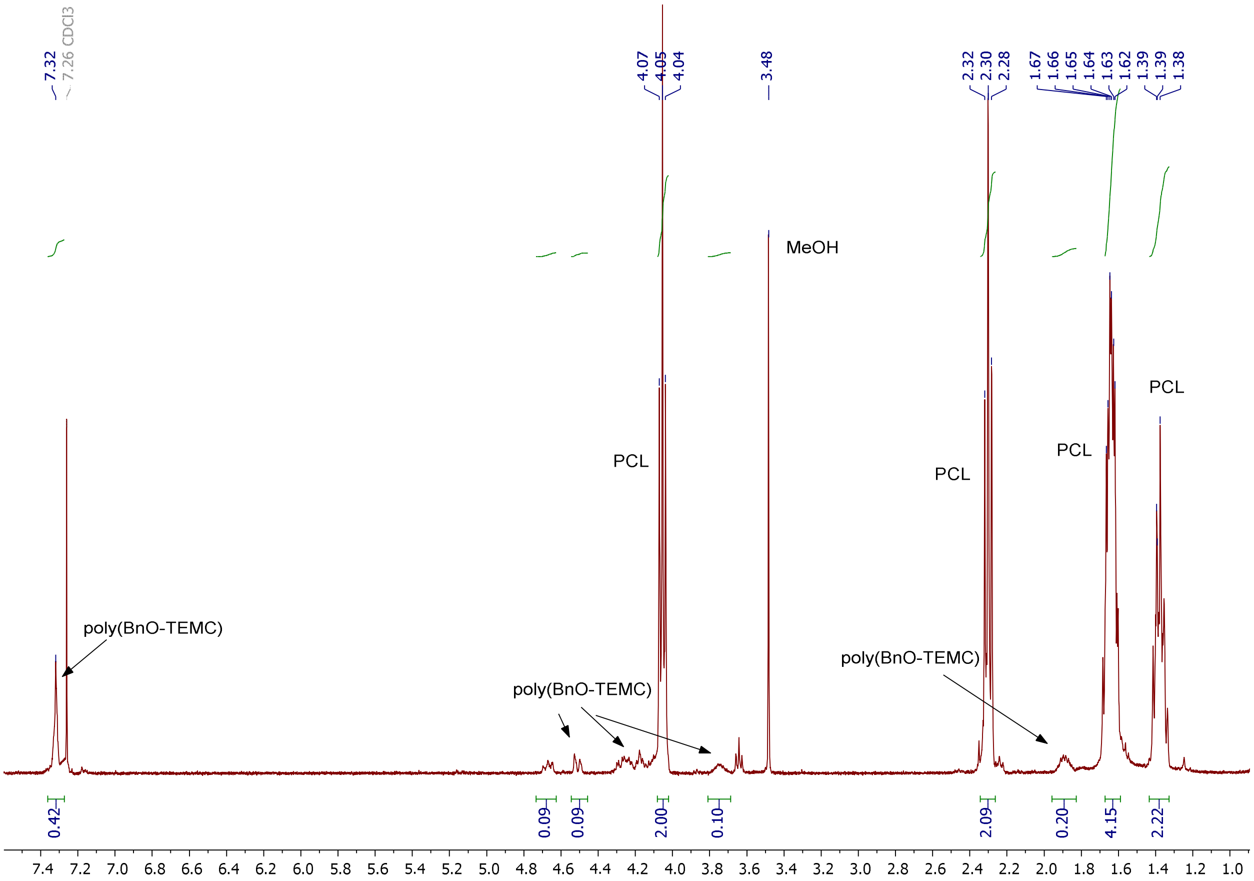

Figure S11. ^{1}H NMR spectrum of poly(BnO-TEMC-*co*-CL) obtained by Sn(Oct)$_2$-catalyzed ROP in toluene solution at 100°C for (Table 1, entry 3).

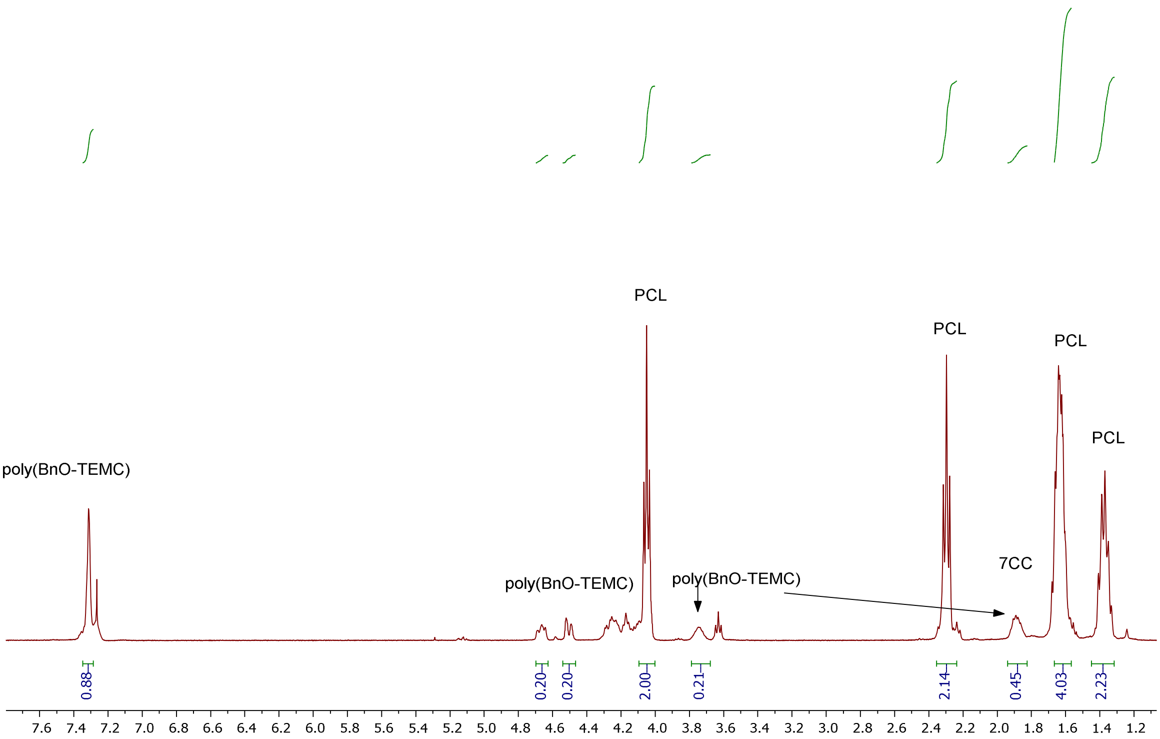

Figure S12. ^{1}H NMR spectrum of poly(BnO-TEMC-*co*-CL) obtained by Sn(Oct)$_2$-catalyzed ROP in toluene solution at 100°C for (Table 1, entry 4).

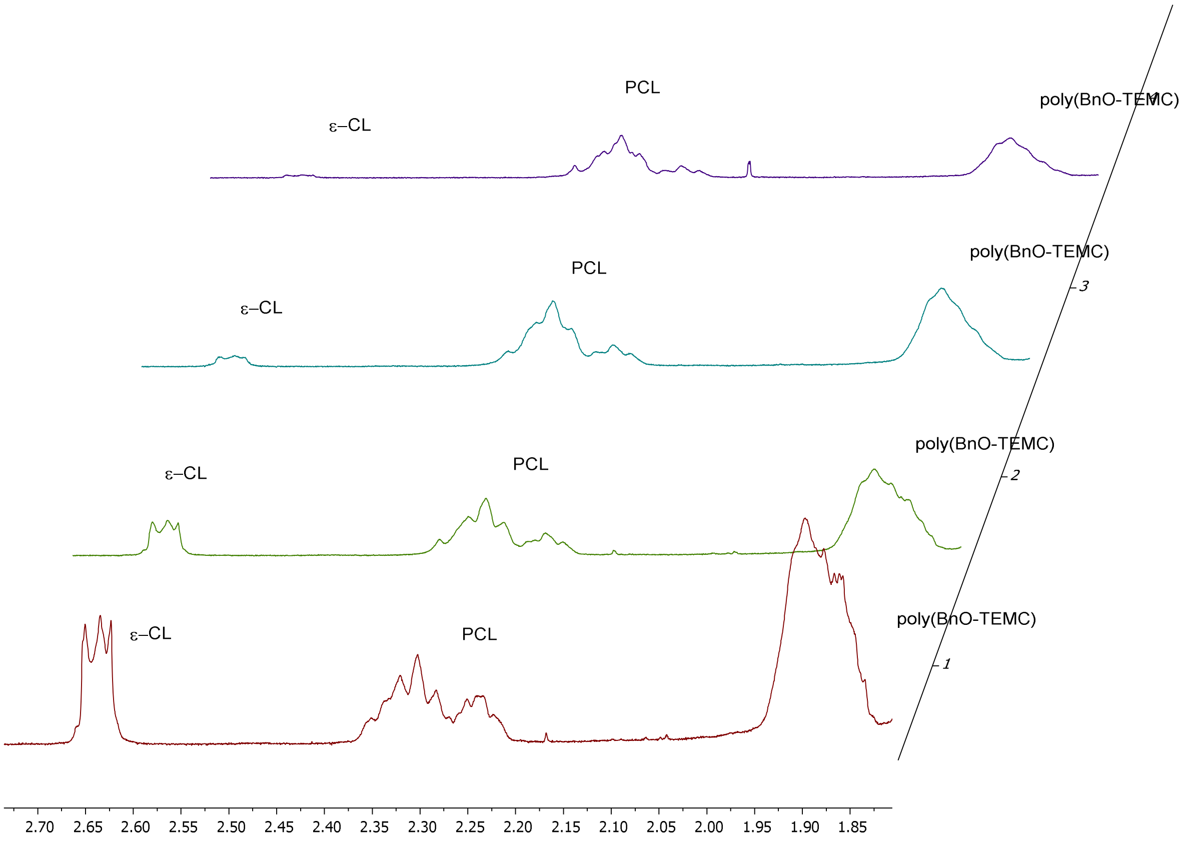

Figure S13. ^{1}H NMR monitoring of ε-CL and β-BnO-TEMC copolymerization conversion (Table 1, entry 5).

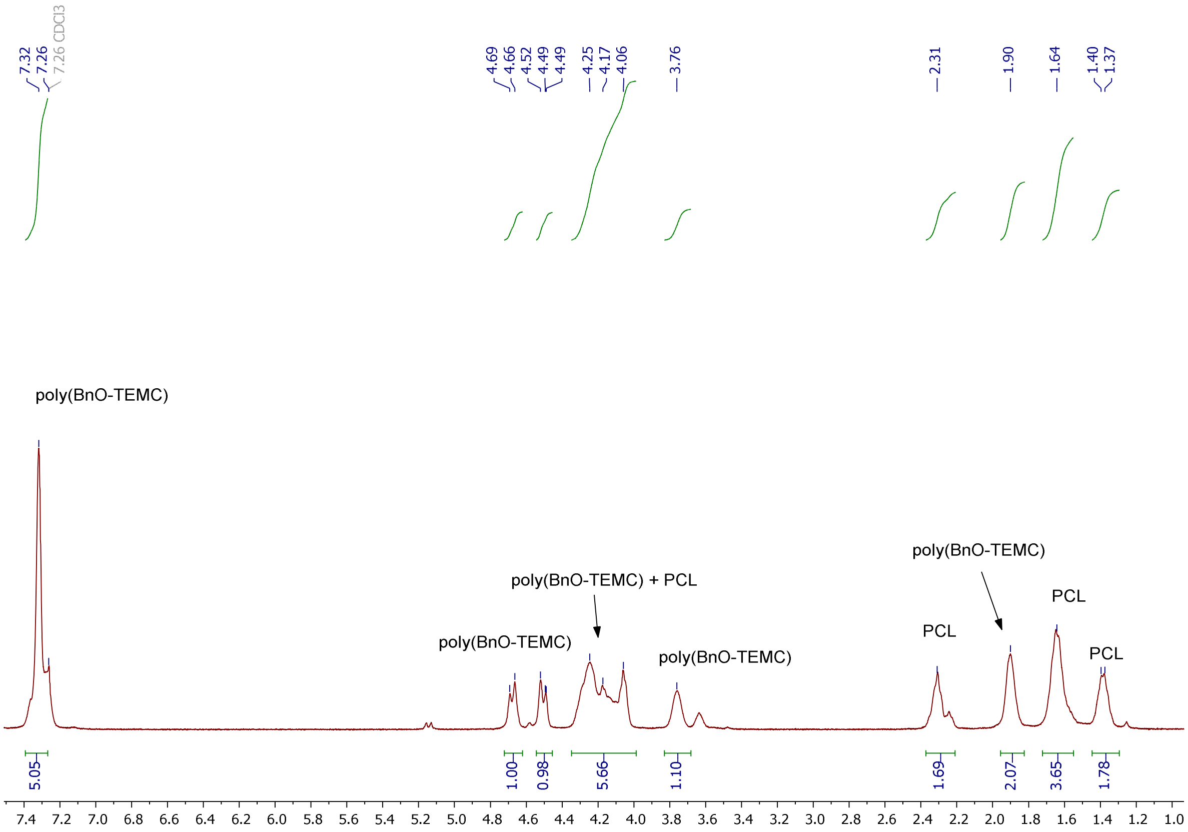

Figure S14. ^{1}H NMR spectrum of poly(BnO-TEMC-*co*-CL) obtained by $Sn(Oct)_2$-catalyzed ROP in toluene solution at 100°C for (Table 1, entry 5).

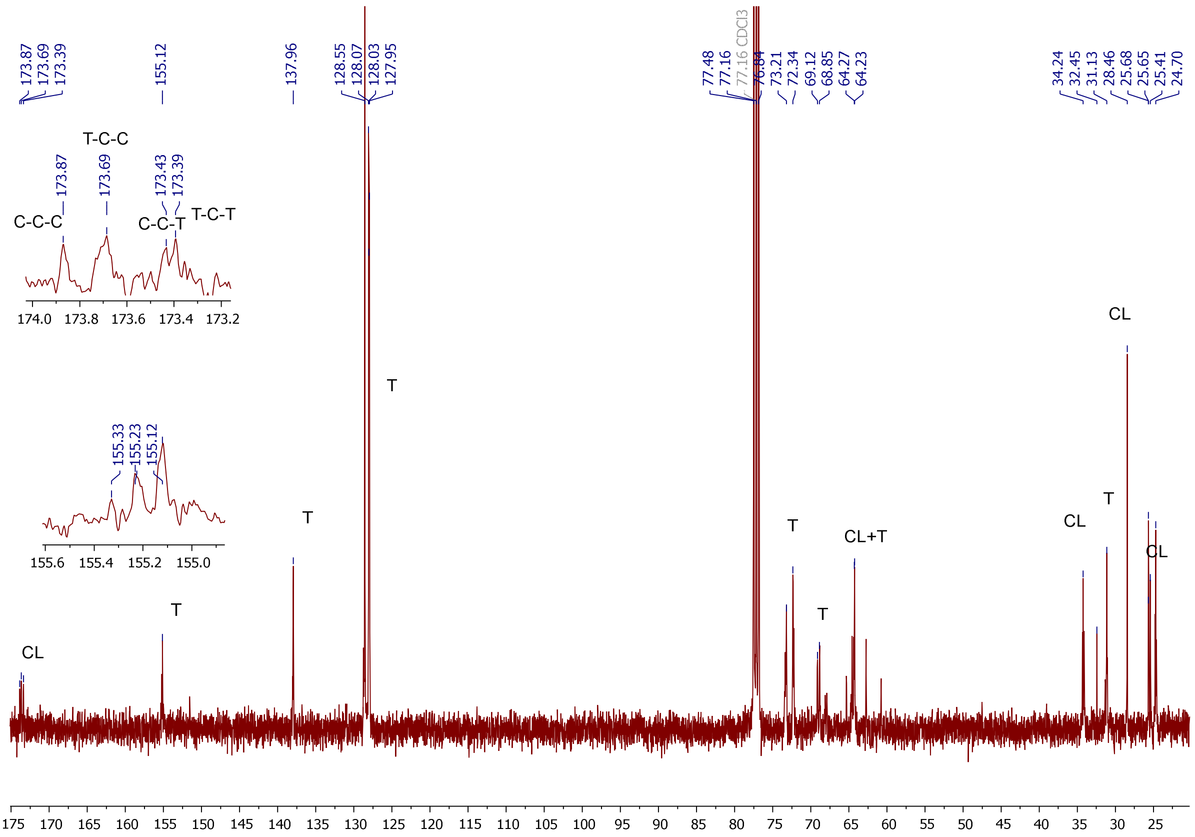

Figure S15. ^{13}C NMR spectrum of poly(BnO-TEMC-*co*-CL) obtained by $Sn(Oct)_2$-catalyzed ROP in toluene solution at 100°C for (Table 1, entry 5).

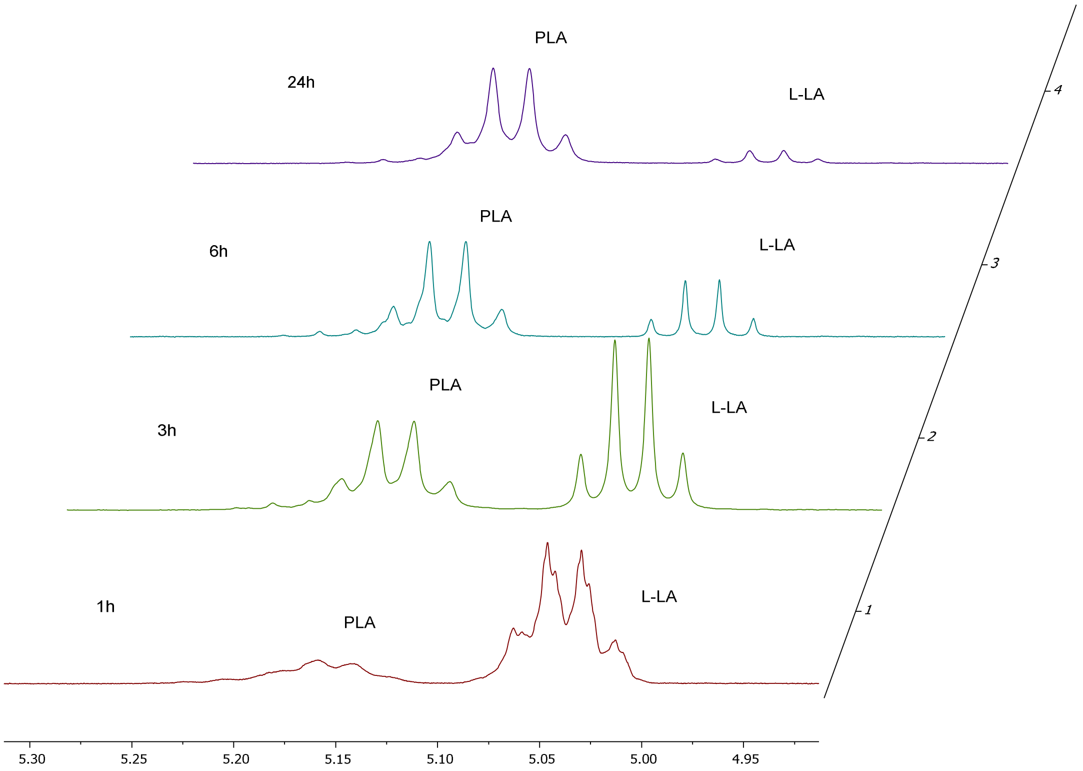

Figure S16. ^{1}H NMR monitoring of L-LA polymerization conversion (Table 1, entry 6).

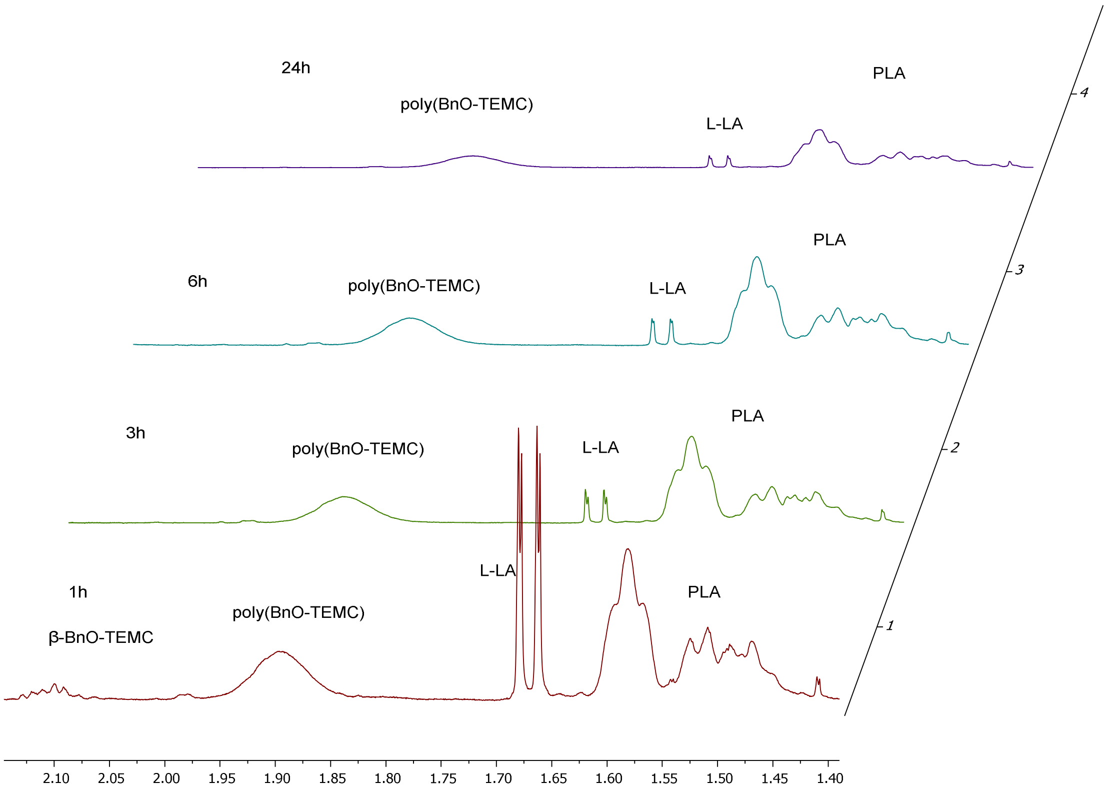

Figure S17. ^{1}H NMR monitoring of L-LA and β-BnO-TEMC copolymerization conversion (Table 1, entry 7).

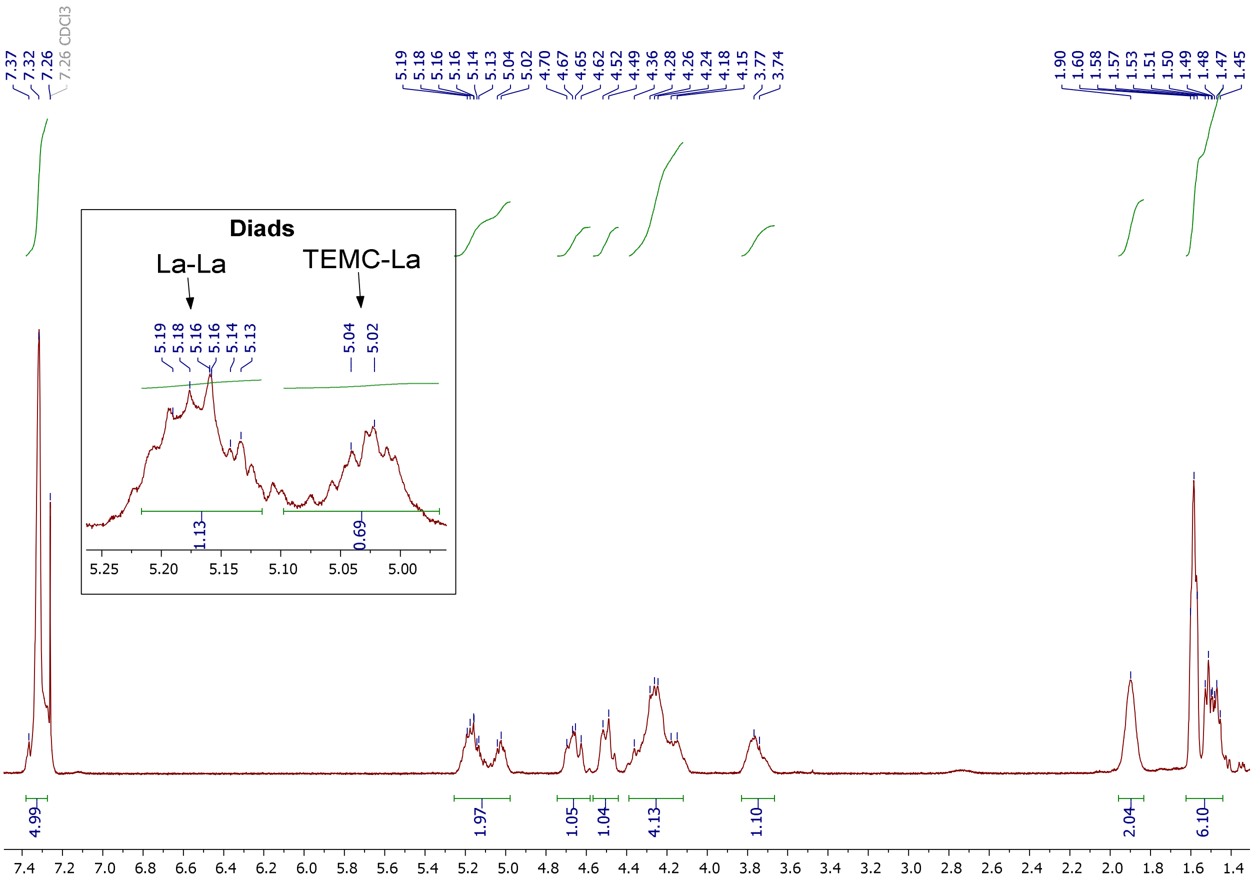

Figure S18. ^{1}H NMR spectrum of poly(BnO-TEMC-*co*-LA) obtained by $Sn(Oct)_2$-catalyzed ROP in toluene solution at 100°C for (Table 1, entry 7).

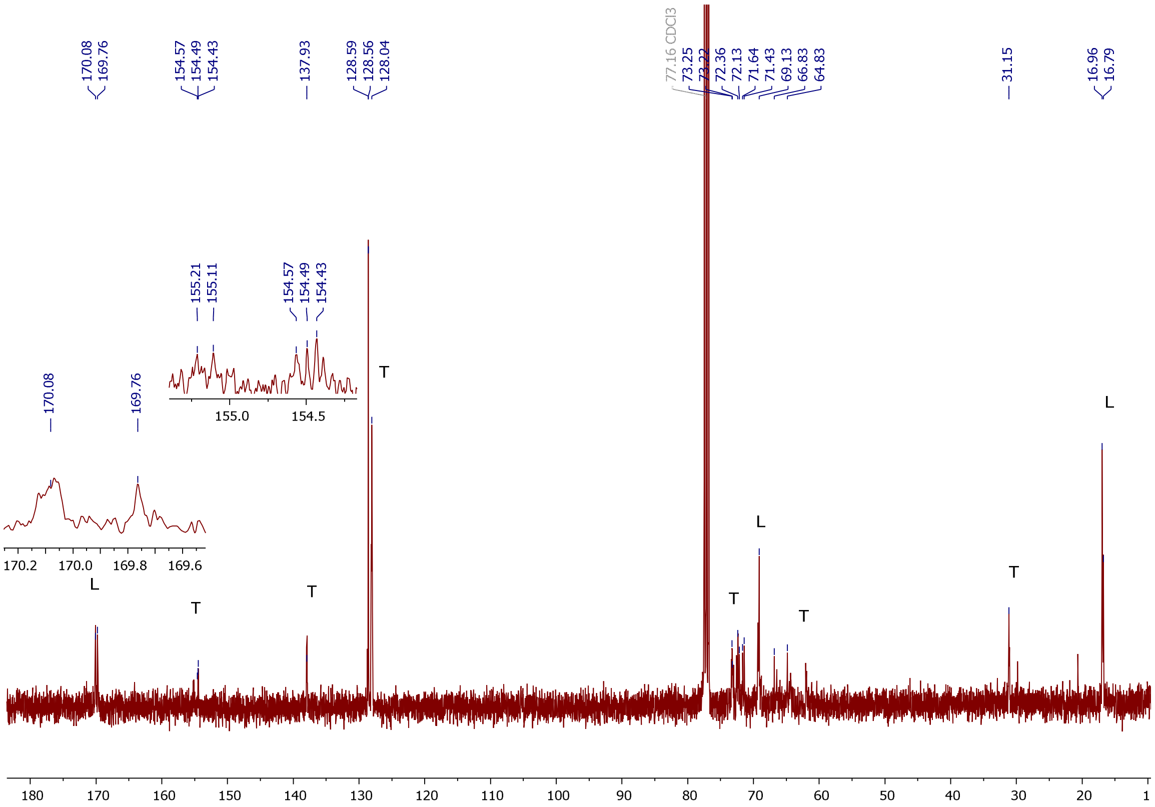

Figure S19. ^{13}C NMR spectrum of poly(BnO-TEMC-*co*-LA) obtained by $Sn(Oct)_2$-catalyzed ROP in toluene solution at 100°C for (Table 1, entry 7).

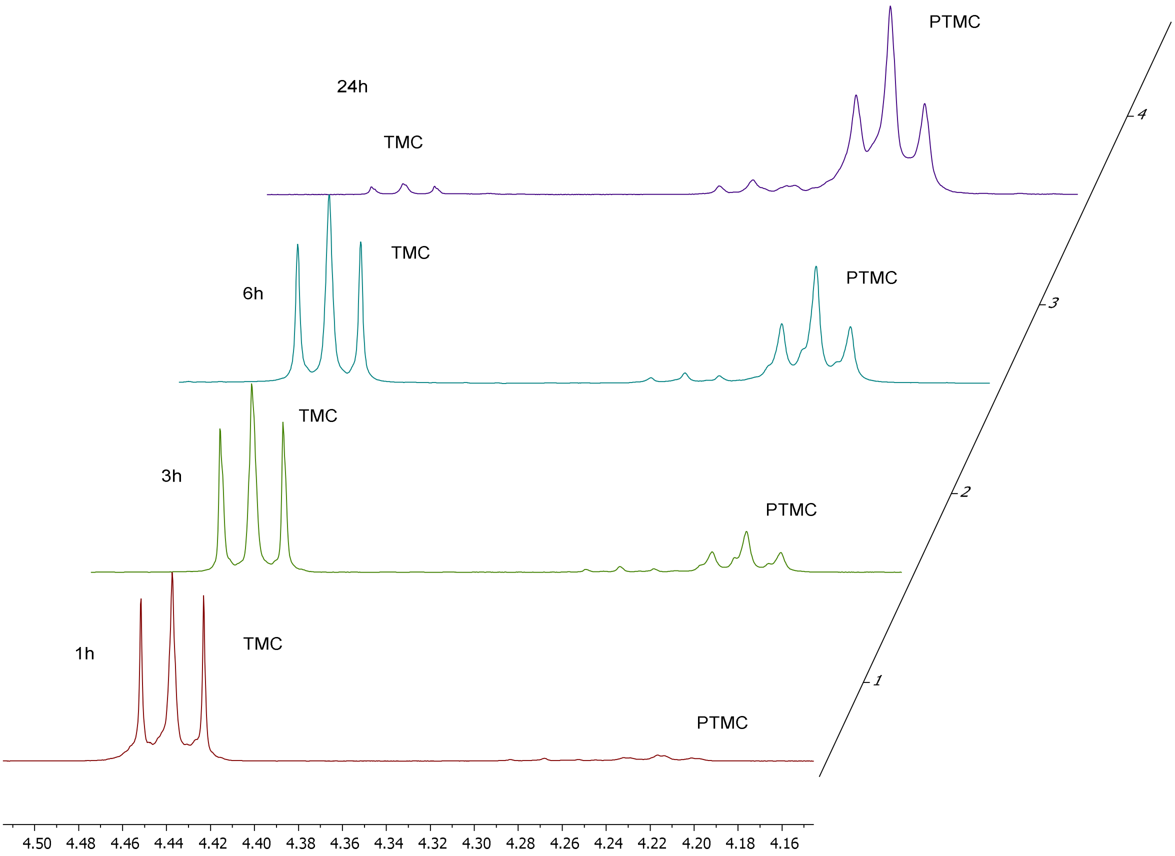

Figure S20. ^{1}H NMR monitoring of TMC polymerization conversion (Table 1, entry 8).

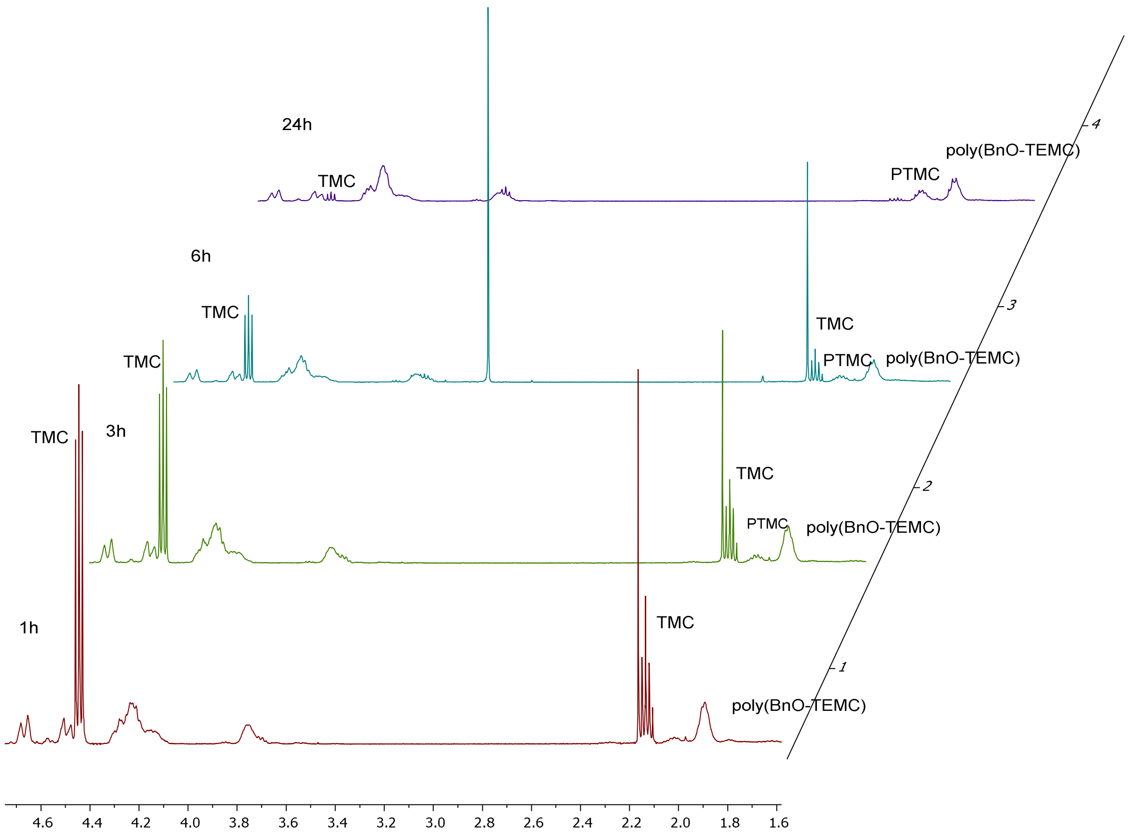

Figure S21. ^{1}H NMR monitoring of TMC and β-BnO-TEMC copolymerization conversion (Table 1, entry 9).

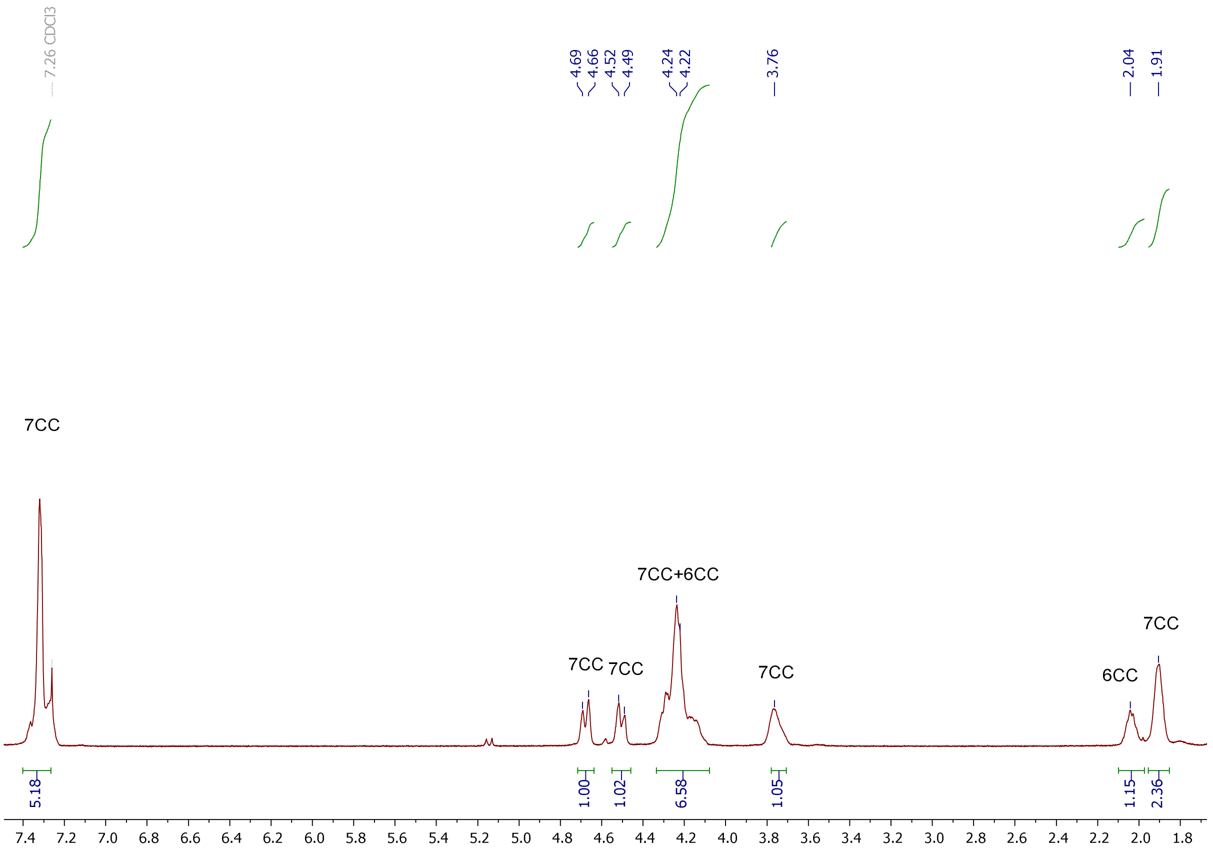

Figure S22. ^{1}H NMR spectrum of poly(BnO-TEMC-*co*-TMC) obtained by $Sn(Oct)_2$-catalyzed ROP in toluene solution at 100°C for (Table 1, entry 9).

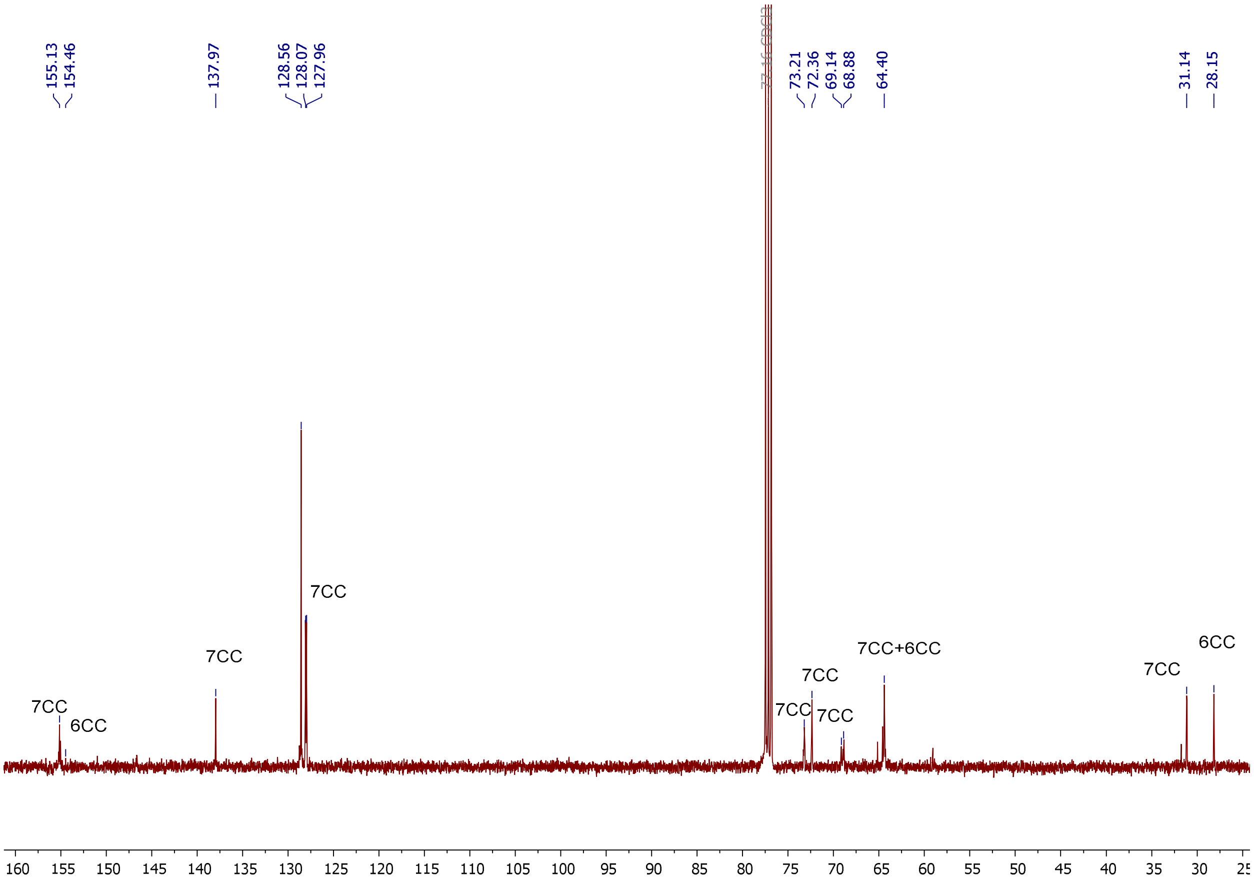

Figure S23. ^{13}H NMR spectrum of poly(BnO-TEMC-*co*-TMC) obtained by Sn(Oct)$_2$-catalyzed ROP in toluene solution at 100°C for (Table 1, entry 9).

Normalized SEC curves of polymers

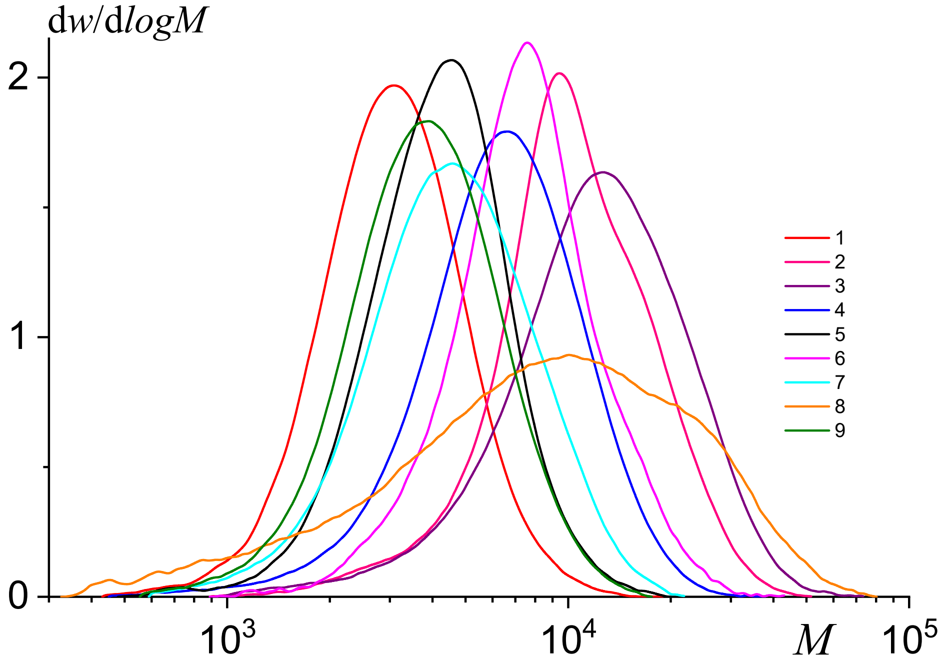

Figure S24. Normalized GPC curves of the samples for entries 1-9 (Table 1).
